# Skeletal muscle alterations in tachycardia-induced heart failure are linked to deficient natriuretic peptide signalling and are attenuated by RAS-/NEP-inhibition

**DOI:** 10.1371/journal.pone.0225937

**Published:** 2019-12-04

**Authors:** Alexander Dietl, Ingrid Winkel, Gabriela Pietrzyk, Michael Paulus, Astrid Bruckmann, Josef A. Schröder, Samuel Sossalla, Andreas Luchner, Lars S. Maier, Christoph Birner

**Affiliations:** 1 Department of Internal Medicine II, University Hospital Regensburg, Regensburg, Germany; 2 Institute of Pathology, University of Regensburg, Regensburg, Germany; 3 Department of Biochemistry I, University of Regensburg, Regensburg, Germany; 4 Electron Microscopy Core Facility (Emeritus), Institute for Pathology, University Hospital Regensburg, Regensburg, Germany; 5 Klinik fuer Kardiologie, Krankenhaus der Barmherzigen Brueder, Regensburg, Germany; 6 Department of Internal Medicine I, Klinikum St. Marien, Amberg, Germany; Temple University, UNITED STATES

## Abstract

**Background:**

Heart failure induced cachexia is highly prevalent. Insights into disease progression are lacking.

**Methods:**

Early state of left ventricular dysfunction (ELVD) and symptomatic systolic heart failure (HF) were both induced in rabbits by tachypacing. Tissue of limb muscle (LM) was subjected to histologic assessment. For unbiased characterisation of early and late myopathy, a proteomic approach followed by computational pathway-analyses was performed and combined with pathway-focused gene expression analyses. Specimen of thoracic diaphragm (TD) served as control for inactivity-induced skeletal muscle alterations. In a subsequent study, inhibition of the renin-angiotensin-system and neprilysin (RAS-/NEP) was compared to placebo.

**Results:**

HF was accompanied by loss of protein content (8.7±0.4% vs. 7.0±0.5%, mean±SEM, control vs. HF, p<0.01) and a slow-to-fast fibre type switch, establishing hallmarks of cachexia. In ELVD, the enzymatic set-up of LM and TD shifted to a catabolic state. A disturbed malate-aspartate shuttle went well with increased enzymes of glycolysis, forming the enzymatic basis for enforced anoxic energy regeneration. The histological findings and the pathway analysis of metabolic results drew the picture of suppressed PGC-1α signalling, linked to the natriuretic peptide system. In HF, natriuretic peptide signalling was desensitised, as confirmed by an increase in the ratio of serum BNP to tissue cGMP (57.0±18.6pg/ml/nM/ml vs. 165.8±16.76pg/ml/nM/ml, p<0.05) and a reduced expression of natriuretic peptide receptor-A. In HF, combined RAS-/NEP-inhibition prevented from loss in protein content (8.7±0.3% vs. 6.0±0.6% vs. 8.3±0.9%, Baseline vs. HF-Placebo vs. HF-RAS/NEP, p<0.05 Baseline vs. HF-Placebo, p = 0.7 Baseline vs. HF-RAS/NEP).

**Conclusions:**

Tachypacing-induced heart failure entails a generalised myopathy, preceding systolic dysfunction. The characterisation of “pre-cachectic” state and its progression is feasible. Early enzymatic alterations of LM depict a catabolic state, rendering LM prone to futile substrate metabolism. A combined RAS-/NEP-inhibition ameliorates cardiac-induced myopathy independent of systolic function, which could be linked to stabilised natriuretic peptide/cGMP/PGC-1α signalling.

## Introduction

Systolic heart failure remains a major healthcare challenge[1]. The mortality remains unacceptably high, albeit the progression of disease can be protracted by improved therapeutic opportunities[2]. During the gained time period between diagnosis and death, advances in pharmacological and interventional treatment helped to relieve patients’ dyspnoea and phases of decompensation[3,4]. As these canonical hallmarks of systolic heart failure can be better controlled than 20 years ago, the consequences of heart failure induced systemic metabolic failure have come to the fore of patients’ symptoms[5]. Particularly, skeletal muscle wasting is frequent, limits patients’ physical capacity and predicts independently death in heart failure[6]. Despite extensive scientific efforts, its end-stage “cachexia” can hardly be addressed therapeutically. Accordingly, strategies for early diagnosis and prevention were emphasised by consensus statements and a “pre-cachectic” state was defined and subjected to further studies[7]. Unfortunately, an animal model showing reproducibly and stable an early state of cardiac-induced myopathy and its steady progression in systolic heart failure is lacking[8]. Therefore, we set out to evaluate, whether the tachypacing-heart failure model[9,10] entails progressive myopathy. As a very early, generalized myopathy similar to humans could be established, the enzymatic set-up was characterised by a multi-omics approach, applying pathway-focused gene expression analysis[11] and proteomic methods[12,13]. Pathway analyses gave hints for a failing link between natriuretic peptide signalling and peroxisome-proliferator-activated-receptor-γ-coactivator-1-α (PGC-1α), in line with previous in-vitro and in-vivo data under physiologic conditions[14]. Therefore, we speculated about a beneficial effect to myopathy by counterbalancing the desensitised natriuretic peptide signalling in heart failure[15] and performed subsequently a pharmacological intervention: combined inhibition of the renin-angiotensin system and neprilysin (RAS/NEP) was compared to placebo in heart failure animals.

## Methods

### Animal model

**Fig 1:**
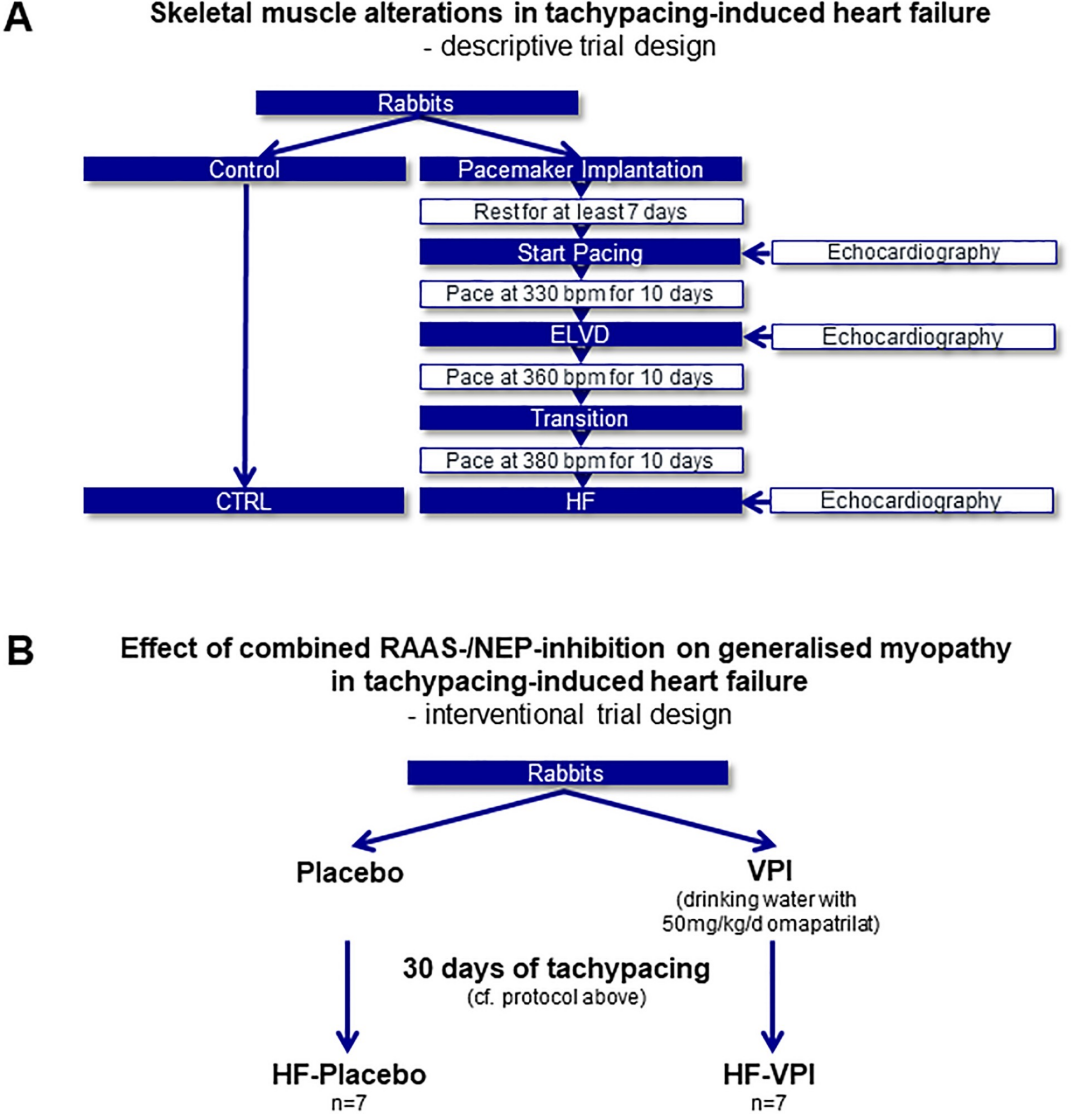
Sequential animal trial designs for first descriptive analyses and the subsequent pharmacological intervention study. (**A**) In a tachypacing-induced heart failure model, skeletal muscle alterations were analysed by histology and a multi-omics approach. The results generated the hypothesis, that combined RAS-NEP-inhibition could exert a potential beneficial effect on myopathy in heart failure. To scrutinise the hypothesis, a pharmacological interventional study was designed and performed (**B**). *bpm*: *beats per minute*. *CTRL*: *control*. *ELVD*: *early left ventricular dysfunction*. *HF*: *heart failure*. *RAS-NEP-inhibition*: *combined inhibition of the renin-angiotensin system and neprilysin*. *VPI*: *vasopeptidase inhibitor omapatrilat*, *combined RAS/NEP-inhibitor*.

All studies were approved by the institutional and governmental animal care committee (Regierung der Oberpfalz, Germany; University of Regensburg, Germany). Male rabbits (chinchilla bastard) were acquired from Charles River Laboratories and housed under standard conditions (12:12h light:dark rhythm) with regular, unrestricted diet. For the first descriptive study characterising skeletal muscle alterations in tachycardia-induced heart failure (Fig 1A), 11 rabbits underwent implantation of a programmable cardiac pacemaker (Medtronic Minix 8340, Minneapolis, MN, USA, or Vitatron Model 810, Dieren, NL) as previously described[9]. In brief, ketamine (60mg/kg) and xylazine (5mg/kg) were given as intramuscular bolus injection to establish anaesthesia and were administered intravenously during the procedure according to the animal’s vital signs. The pacemaker lead was inserted into the right internal jugular vein, passed to the right ventricular apex under fluoroscopic guidance and fixed by screwing it into the myocardial tissue. The device was implanted subcutaneously into the right abdominal wall. A standardized pharmacological protocol was used in the early post-surgery period (rimadyl 4mg/kg s.c.; baytril 5mg/kg s.c. for 3 days). After at least 10days at rest, a V00-Pacing mode was programmed and conducted tachypacing for a total of 30days, increasing stepwise the stimulation frequencies at 10 days intervals (330beats/min; 360 beats/min; 380beats/min). 4 animals were euthanized after 10 days of pacing, resulting in early left ventricular dysfunction (ELVD group). Further 7 animals went through 30 days of incremental pacing, showing signs of heart failure (HF group), as ascites and pleural effusion. 6 untreated animals served as controls (CTRL group).

In the subsequent interventional trial (Fig 1B), a combined RAS-/NEP-inhibition was administered as previously described[12]. Since LCZ696 was not yet available for research purposes at that time, the vasopeptidase inhibitor omapatrilat was used. In animal experiments, it has been given by intravenous bolus application for the evaluation of acute drug effects[16] and by gavage[17] or dissolved in drinking water[18,19] or food [20] for long term administration. Due to anatomical features of rabbits, gavage is accompanied by a higher risk of trauma to oesophageal and gastric lining and aspiration pneumonia than in rats or mice[21]. As omapatrilat is dissolvable in water, has good bioavailability, high distribution volume and long half-life[22], it was administered via drinking water in order to avoid injury to the animals by repeated gavage. Adequate concentration of omapatrilat was validated in our previous studies[12,23].

Rabbits’ fluid intake was monitored on a daily basis for each animal individually and drinking water was freshly prepared for each animal every day. In order to achieve a uniform dosage, an amount of stock solution of omapatrilat was added to the fresh drinking water in such a manner, that a dosage of 50mg/kg/d was achieved in an amount of drinking water, which was equal to the previous average daily drinking amount of that individual animal. Together, drinking water of animals undergoing 30days of tachypacing was either substituted with omapatrilat (HF-VPI group, n = 7), or remained untreated (HF-placebo group, n = 7). At baseline (BL) and at the end of the protocol (HF), conscious arterial pressure was measured invasively: an intravascular cannula was inserted into the medial ear artery. An electronic pressure transducer (P23XL; Siemens, Munich, Germany) and a recorder (Hellige, Freiburg, Germany) were employed to monitor constantly the blood pressure. 5 animals not undergoing tachypacing served as controls (CTRL-2; n = 5). After euthanasia by pentobarbital injection, tissue of the M. quadriceps femoris (limb muscle, LM) and the muscular part of the thoracic diaphragm (TD) was rapidly harvested, deep-frozen in liquid nitrogen and stored at -80°C.

### Echocardiography

Echocardiographic assessment was performed under moderate sedation (acepromazine, 0.07mg/kg) and pacing was intermitted temporarily as previously described[9,10,12]. A HP Sonos 5500 equipped with a 12MHz transducer (Philips Electronics, Eindhoven, the Netherlands) was applied to measure left ventricular end-diastolic (LVEDD) and end-systolic diameter (LVESD) by two-dimensionally guided M-Mode in the parasternal long axis in accordance to the current European guidelines[24]. Systolic function was determined as fractional shortening (FS), because FS provides reliable information in heart disease without regional wall motion abnormalities according to current guidelines[24], measurement of ejection fraction is hardly feasible in rabbits [25] and the necessary longer examination time for biplane measurement from an apical view under sedation entails the relevant risk of lethal respiratory insufficiency in end-stage HF rabbits. FS was calculated as FS = (LVEDD-LVESD)/LVEDD.

### Determination of muscle fibre types

**Fig 2:**
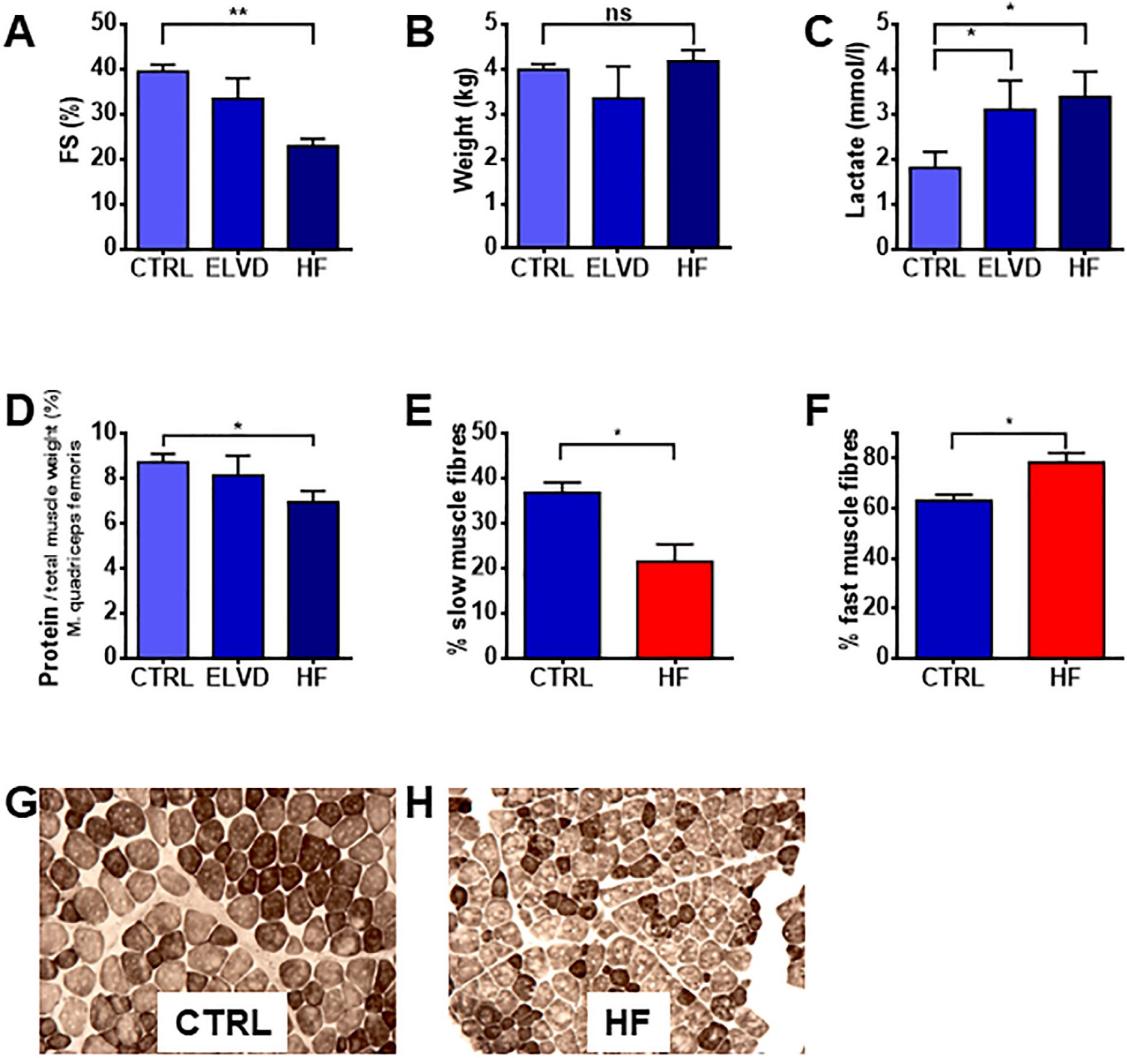
Tachypacing leads to systolic heart failure and signs of sarcopenia. Rapid ventricular pacing caused left ventricular dysfunction (**A**). Body weight remained stable (**B**), due to ascites and pleural effusion in HF. Heart failure syndrome was accompanied by raising lactate levels (**C**), decreased protein content of LM (**D**) and a slow-to-fast fibre type switch (**E, F**), which is depicted by representative slices (**G, H**): after specific inhibition of ATPase by alkali (type I fibres) and acid (type II fibres), ATPase staining shows dark slow and light fast fibres. *A-C*: *paired t-test n = 5 for each group; D*: *paired t-test n = 3 for each group; E*, *F*: *Welch’s unequal variances t-test*. *p<0.*05*, **p<0.*01*. *n(CTRL/HF) = 6/3*. *FS*: *fractional shortening*. *CTRL*: *control*. *ELVD*: *early left ventricular dysfunction*. *HF*: *heart failure*. *LM*: *limb muscle*.

For optimizing cryo-sections, frozen tissue of skeletal muscles was treated as previously described[26]. Briefly, tissue was thawed, dehydrated, again frozen in 2-methylbutanol and sliced (Leica Biosystems, Nussloch, Germany). To distinguish muscle fibre types, differences of the actomyosin ATPase were determined by exposing sections to alkali or acid before staining for ATPase[27]. Acid pre-incubation inhibits the actomyosin ATPase activity in fast (type II), but not slow (type I) fibre types. In contrast, basic pre-incubation inhibits actomyosin ATPase in slow (type I), but not fast fibre types. Chemicals were acquired from Sigma, St. Louis, MO, USA. After 5 minutes of fixation (formaldehyde 2%, sodium cacodylate 0.19M, CaCl_2_ 0.07M, sucrose 0.34M, pH 7.6) and 1 minute of washing in rinse solution (18mM CaCl_2_, tris(hydroxymethyl)aminomethane 100mM, pH 7.8), slides were pre-incubated in alkaline solution (CaCl_2_ 18mM, 2-amino-2-methyl-1-propanol 0.1M, pH 10.4) for 15min. After washing them twice, they were incubated for 60 minutes (ATP 2.7mM, KCl 50mM, CaCl_2_ 18mM, pH 9.4, 37°C), rinsed three times for 30 seconds each (CaCl_2_ 1%w/v) and exposed to cobalt chloride 2% (w/v) for 3 minutes. After washing them again 4-times for 30seconds each (2-amino-2-methyl-1-propanol 0.1M, pH 9.4), they were incubated in ammonium sulfide 1%w/v and washed in bi-distilled water for 4 minutes. Afterwards, slides were dehydrated in graded ethanol, cleared in xylol and embedded in entellan (Merck, Darmstadt, Germany). For exposing tissue to acid, the same procedures were basically employed except for the fact, that there was no fixation. For acidic pre-incubation, a different solution was used (CaCl_2_ 18mM, potassium acetate 50mM, pH 4.35) for 25 minutes. The stained tissue was visualized by microscopy (Zeiss, Oberkochen, Germany). For quantitative analysis comprising specimen of all animals, nine sections were done from each individual. After acid pre-incubation, only type I fibres stained darkly (Fig 2G and 2H). The area of darkly (type I) and lightly (type II) stained fibres was quantified by ImageJ (version 1.48v).

### Isolation of mitochondria

For the isolation of mitochondria, a commercially available kit was used according to manufacturer’s instructions (NBP2-29448, Novus Biologicals, Littleton, CO, USA). Results were checked for sufficiency of isolation by western blotting and electron microscopy.

### Electron microscopy

Isolated mitochondria samples were incubated in Karnovsky-fixative. Transmission electron microscopy was performed as previously described[9,12] using an EFTEM LEO912AB (Zeiss, Oberkochen, Germany) equipped with an 1kx1k pixel side-entry mounted CCD camera controlled by the iTEM software (OSIS, Muenster, Germany). The analysis was focused on number of mitochondria and their integrity.

### qPCR

mRNA was extracted from TD and LM samples utilizing a commercially available kit (RNeasy, Qiagen, Venlo, Netherlands) by following manufacturer’s protocol. RNA yield was photospectrometrically measured at 260nm. Expression levels of natriuretic peptide receptors A, B and C and peroxisome proliferator-activated receptor gamma coactivator 1-alpha (PGC-1α) were analysed using Custom TaqMan Assays provided by ThermoFisherScientific, Waltham, MA, USA, (NPR-A = XM002715497; NPRB = XM008268071; NPRC = XM008262100, GAPDH = OC03823402_g1; PGC-1α = XP002709423). GAPDH was used as housekeeping gene. All analyses were run in triplicate.

### Pathway-focused gene expression analysis

For pathway-focused gene expression analysis, assays targeting genes relevant to the pathways of interest, which resulted from the proteome analysis, were applied. Commercially available array kits (Qiagen) were customized for rabbits based on RT^2^ Profiler PCR Arrays targeting mitochondria (PANZ-087Z), PPAR signalling (PANZ-149Z), and fatty acid metabolism (PANZ-007Z). The detailed gene lists are depicted in the **Supplementary material** S1 File (**Tables A, B, C**). qPCR was carried out using a ViiA7 Real-Time PCR System equipped with ViiA7 RUO software (ThermoFisherScientific) and the RT^2^ SYBR Green qPCR master mix (Qiagen) according to the RT^2^ Profiler PCR Array instructions. The results were analysed as described[11]: microarray data were normalised against the house keeping genes, as the ΔCT for each gene in the plate was computed. The RT^2^ PCR array data analysis web portal (http://saweb2.sabiosciences.com/pcr/arrayanalysis.php, accessed 03/2018) provided the tools for further descriptive statistics, performing tests and plotting the results. A two-sided error probability below 0.05 was deemed to be significant.

### Protein extraction

Frozen LM and TD tissue was ground up under liquid nitrogen and transferred to lysis buffer (urea 7M, thiourea 2M, Tris 30mM, 3-((3-cholamidopropyl)dimethylammonium)-1-propanesulfonate 4%w/v, Aprotinin bovine lung 0.045–0.315TIU/ml, pH 8.5). Alternatively, when analysing particularly mitochondrial proteins, the pellet containing isolated mitochondria was resuspended in lysis buffer. Overall protein content was determined by a bicinchoninic acid assay (Sigma) according to manufacturer’s instructions.

### 2-D fluorescence difference gel electrophoresis (2-D DIGE)

Protein lysates of LM and TD tissue and, subsequently, of isolated LM and TD mitochondria were subjected to two-dimensional fluorescence difference in gel electrophoresis (2D-DIGE). To precisely load 2-D-gels, the protein concentration was quantified by 2D Quant Kit (GE Healthcare, Chalfont St Giles, UK). A pH between 8.0 and 9.0 was carefully titrated. The 3Dye 2D DIGE kit (Lumiprobe, Hannover, Germany) containing 3 different cyanine dyes was used to label proteins according to manufacturer’s instructions. Lysates of all specimens were pooled to an internal standard. On each gel, 3 samples were run simultaneously: 2 actual probes and the internal standard. Controls and disease states were not pooled, but analysed as biological individuals. 2-D gel electrophoresis was performed as previously reported[9]: the samples were standardized by containing protein weight (50μg). 3 samples labelled with Cy2, 3 or 5 were combined and transferred to 350μl rehydration buffer (7M urea, 2M thiourea, 4% CHAPS, 1% Serdolit MB-1 p.A. (SERVA, Heidelberg, Germany), 1.5% DeStreak Reagent (Amersham Biosciences, Uppsala, Sweden), 0.5% Pharmalyte). For the first dimension electrophoresis, immobilized pH gradient strips (IPG 3-10NL, 18cm, GE Healthcare) were used on the Ettan IPGphor 3 Isoelectric Focusing Unit (GE): starting with an active rehydration (16h, 50V, 0.05mA/strip), the protocol provided an incremental increase of voltage (500V for 135min, 1000V for 90min, rapid voltage ramping to 8000V for 60min, 8000V for 240min; T = 20°C). Afterwards, the strips were equilibrated (1% (w/v) DTT, 6M urea, 30% (w/v) glycerol, 2% (w/v) SDS, 0.05M Tris-HCl buffer, pH = 8.8) for 15min and once again in a similar solution, containing iodoacetamide 8% (w/v) instead of DTT. For the second dimension, polyacrylamide gels were cast (12.5 (v/v) polyacrylamide, 3.3% w/w crosslinking, 26x20x0.1cm) between low-fluorescence glass plates (DALTsix Gel Caster, GE). The vertical electrophoresis was accomplished in an Ettan DALTsix Electrophoresis Unit using a PowerSupply EPS 601(GE; U = 600V; I = 400mA; 17h; 10°C). Gels were scanned by ChemiDoc MP (Cy2 λ_ex_/ λ_em_ 490/518nm; Cy3 λ_ex_/ λ_em_ 545/577nm; Cy5 λ_ex_/ λ_em_ 645/ 675nm) and analysed using dedicated software (MELANIE, version 7.0.6, Geneva Bioinformatics, Geneva, Switzerland), performing spot detection, in-gel normalisation, gel-to-gel matching and statistical analysis. The differences between subgroups were tested for significance by one-way analysis of variance (ANOVA).

### Protein identification

Protein spots showing significant differential normalised intensity were excised manually and sequentially identified by peptide-mass fingerprinting. As the protein amount on DIGE labelled gels was small, spot intensity was too low for an unambiguous detection “by eye” for manual spot excision. Therefore, for spot picking, gels were loaded with 750μg protein and stained by SyproRuby stain (Sigma) according to manufacturer’s instructions. In brief, gels were fixated (methanol 50%, CH_3_COOH 7%) twice for 30 minutes each and afterwards stained overnight in SyproRuby solution. After washing them in dedicated solution (methanol 10%, CH_3_COOH 7%) for 30 minutes, they were rinsed several times with bi-distilled water. Stained proteins were visualized at a λ_ex_ of 302nm (ChemiDoc MP, BioRad) and manually excised. Proteins were digested by trypsin (sequencing grade, Roche, Penzberg, Germany), extracted from the small pieces of gel, cleansed from interfering substances and identified by matrix-assisted laser desorption/ionisation-tandem mass spectrometry (MALDI-MS/MS) as previously described[9,10,28]. The digest was dissolved in matrix solution and spotted onto the target plates. Mass spectrometry was performed (MALDI-TOF MS/MS, 4800 proteomics analyser running with the v3.5.3 4000 series explorer software, AB Sciex, Framingham, USA) and resulting mass spectra were compared to the NCBI protein database by dedicated software (Mascot, Matrix Science, London, UK). A Mascot score denoting an error probability below 0.05 for protein identification was deemed to be statistically significant.

### Analysis of proteomic data

To assign the biological process and molecular functions defined by the Gene Ontology Consortium[29] to the detected proteins, a comprehensive data search was performed based on the UniProt database[30] for each individual protein using the rabbit proteome (ID: UP000001811, last modified on March 13, 2018). As the rabbit proteome is incompletely annotated, for pathway analysis the coding genes of the proteins were ascertained using the NCBI protein database (accessed 07/2018) and their human orthologs were looked for by the BetterBunny analysis tool (v2.3, updated 11/2015)[31]. Afterwards, the corresponding PANTHER tools[32] were applied (version 13.1, released on February 3, 2018), based on the GO database version 13.1 (released February 3, 2018) as previously described[9].

### Western blot

For western blot, fractions containing isolated mitochondria or lysates of whole tissue LM and TD were stored in RIPA buffer (Tris 50mM, NaCl 150mM, Sodiumdesoxycholat 0.5%, SDS 0.1%, TritonX 1%, EDTA 2mM). They were heated to 95°C for 5 minutes and were subjected to 1-D vertical SDS-gel electrophoresis afterwards (100V, 75minutes) using polyacrylamide gels (7.5 to 12%) and the BioRad Mini PROTEAN Tetra Cell chamber (BioRad, Hercules, CA, USA). For blotting, the Blot Turbo RTA Transfer Kit (BioRad) was used according to manufacturer’s instructions (2.5A, 25V, 3min). Membranes were blocked by 5%-milk powder (Carl Roth, Karlsruhe, Germany) for 1 hour and washed 3-times in in TBS-T (8 minutes). Incubation (overnight, 4°C) was done using the following antibodies of Abcam (Cambridge, UK): VDAC mouse (31kDa), Hsp60 mouse (60kDa), cytochrome C mouse (12kDa) and β-actin mouse (42kDa). Donkey anti-mouse IgG (Abcam) was chosen as secondary antibody (1 hour). Clarity Western ECL substrate (BioRad) was used and chemiluminescence was measured by ChemiDoc MP (BioRad). ImageJ (version 1.48v) was employed for quantitative analysis. To scrutinize the expression levels of the 5 complexes of oxidative phosphorylation, a dedicated antibody cocktail was used (ab110413, abcam, Cambridge, UK), as Anti-PGC-1α (ab106814, abcam) was applied for PGC-1α. Since even small effects of protein regulation of electron transport chain (ETC) complexes and PGC-1α are of major interest regarding to the hypotheses created by 2-D DIGE, stain-free technology was used for normalisation, as previously described[33].

### Enzymatic activities of the mitochondrial respiratory chain

The enzymatic activities of the complexes I-IV were assayed spectrophotometrically. The results were normalized to the activity of the citrate synthase. The activity of complex I (NADH-ubiquinone oxidoreductase) was determined as previously described[34]. Concisely, the oxidation of NADH at a wavelength of 340nm and, for control purposes, the rotenone-insensitive complex I activity were quantified. The activities of complexes II (succinate dehydrogenase) and III (Q-cytochrome-cytochrome-c-oxidoreductase) were measured by a combined assay according to Spinazzi et al[35], who used succinate and cytochrome c as substrates / electron acceptors. The absorbance was measured at 550nm for 3min. The activity of the complex IV-enzyme was quantified by adding a mitochondrial isolate to a solution containing DTT-reduced cytochrome c. The absorbance was measured at a wavelength of 550nm[12]. To determine the activity of citrate synthase, the protocol established by Srere[36] was applied. Briefly, membrane proteins were solubilised by Triton X100. Acetyl-CoA, oxalacetate and DTNB were added. Citrate synthase mediates the reaction of oxalacetate with acetyl-CoA. The free CoA can convert DTNB to TNB, which was assessed by photometry at 412nm (200s). For all experiments a NanoDrop 2000c (ThermoFisher Scientific) was used.

### Natriuretic peptide measurements

After puncture of the marginal ear vein, samples were immediately transferred to chilled Eppendorf-cups on ice for 30min and subsequently centrifuged (10,000g, 4°C, 10min). The supernatant was frozen at -80°C. No freezing or thawing cycles were performed until final measurement. Plasma BNP concentrations were quantified by a competitive enzyme immunoassay (KA1861, Abnova, Taipei City, Taiwan), according to manufacturer’s instructions. The unit pg/ml is displayed. To convert it to recommended SI units of ng/l[37], multiply by 1(ml*ng)/(pg*l). The concentration of BNP’s second messenger cGMP was measured in LM and TD tissue by a competitive enzyme immunoassay (Biotrak cGMP, GE) as recommended by the manufacturer.

### Statistics

Values are shown as mean±standard error of the mean (SEM), if not indicated otherwise. For qPCR, all statistics were calculated for ΔCT values. 95%-Confidence intervals for ΔΔCT-values were computed according to Gauß’ error propagation. The results were potentiated to show the geometric means of fold changes with 95% confidence intervals (CI) according to the 2^(-ΔΔCT)^-method. Testing for significance was computed using the normally distributed log-fold-changes (ΔΔCT) by unpaired student’s t-test.

The fold changes (FC) for ELVD and HF of the ratios of BNP and NPR-A or NPR-B were calculated as:

$$FC\;{\text{BNP/NPR}}_{\left(\text{ELVD}\;\text{or}\;\text{HF}\right)}=\frac{\frac{BNP_{\text{ELVD}\;\text{or}\;\text{HF}}}{BNP_{\text{CTRL}}}}{2^{\Delta \Delta \text{CT}\;(\text{ELVD}\;\text{or}\;\text{HF-CTRL})}}$$

The FC for ELVD and HF of the ratios of cGMP to NPR-A or NPR-B were calculated as:

$$FC\;\text{cGMP}/{\text{NPR}}_{\left(\text{ELVD}\;\text{or}\;\text{HF}\right)}=\frac{\frac{cGMP_{\text{ELVD}\;\text{or}\;\text{HF}}}{{\text{cGMP}}_{\text{CTRL}}}}{2^{\Delta \Delta \text{CT}\;(\text{ELVD}\;\text{or}\;\text{HF-CTRL})}}$$

The according confidence intervals were computed by the Fieller method [38] using the online calculator of GraphPad Software (San Diego, CA, USA)[39].

All statistical analyses were performed using SPSS statistics version 22 (IBM, Armonk, NY, USA) and GraphPad Prism Version 8.2.0. Statistical significance was assigned at a two-sided p-values of less than 0.05.

## Results

### Skeletal muscle in tachypacing-induced heart failure

After 30 days of tachypacing, left ventricular systolic dysfunction was established in the HF group (Fig 2A). The stable body weight (Fig 2B) despite sarcopenia might be due to fluid retention, in as much as the HF animals showed ascites and pleural effusion in the macroscopic post mortem examination. Serum lactate levels were increased in HF (Fig 2C). Regarding muscular alterations, a reduced overall protein content of LM was measured (Fig 2D). Systolic heart failure provoked a slow-to-fast fibre type switch in LM as seen on representative slices (Fig 2G and 2H) and substantiated by quantitative analysis (Fig 2E and 2F). Together, tachypacing-induced heart failure was associated with signs of LM sarcopenia.

### Proteomic screening indicates altered mitochondrial transmembrane transport

**Fig 3:**
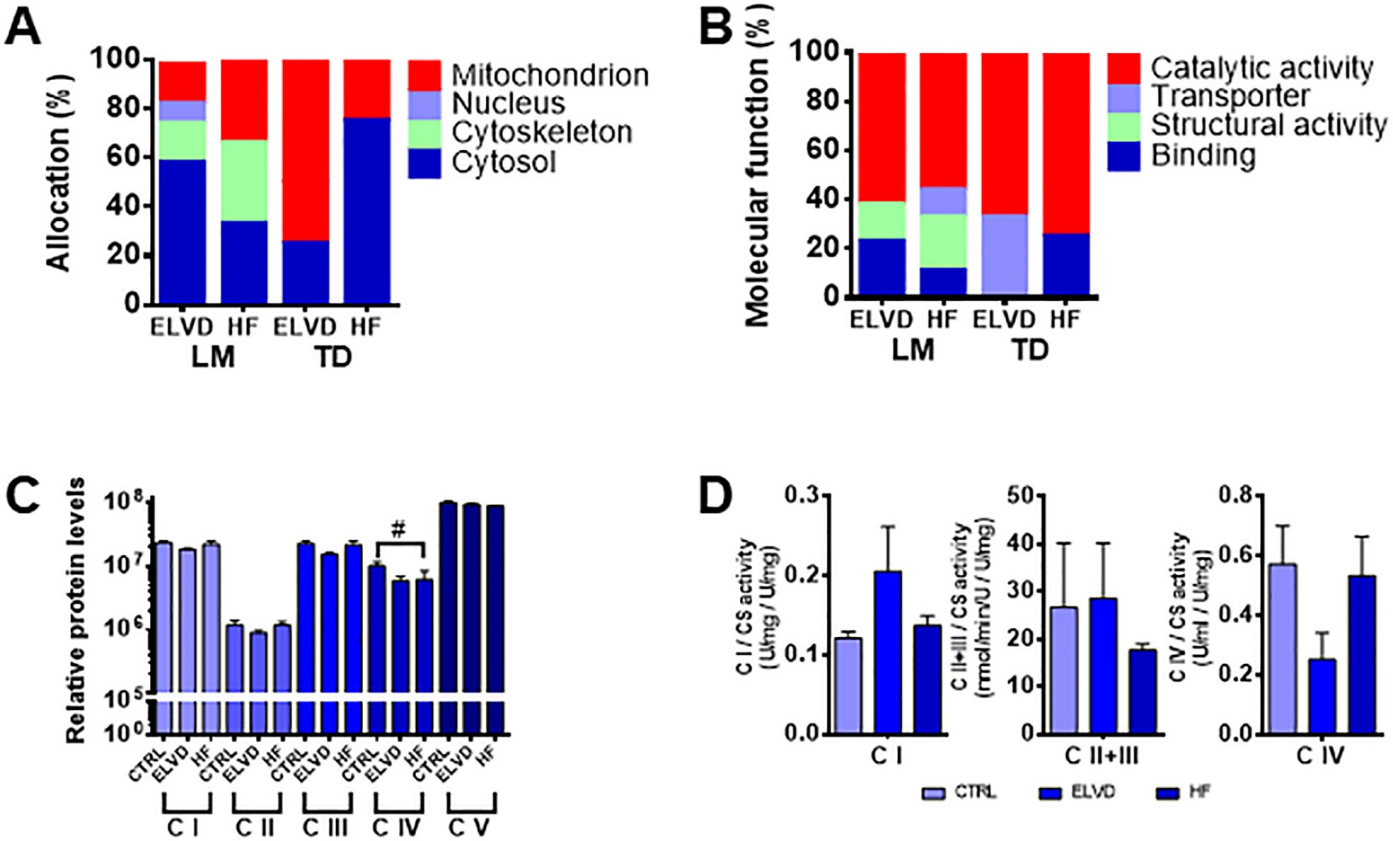
The cytosolic enzymatic set-up of skeletal muscle shifts to catabolic dominance very early in heart failure development. In progressive, tachypacing-induced heart failure, alterations of mainly the cytosolic enzymatic set-up occurred early (**A**) and were characterised by homogenous catabolic dominance in LM and TD (**B**). Despite the cytosolic shift to catabolism, the mitochondrial metabolic enzymes were barely affected: the expression levels of the mitochondrial ETC complexes were not correspondingly more abundant. Complex IV was even less expressed (**C**), which did however not translate to an altered enzymatic activity (**D**). *A*, *B*: *Allocation (A) and molecular function (B) of the proteins*, *whose concentrations were altered in ELVD and HF compared to CTRL*. *C*: *#p<0*.*05 Bonferroni post-test for p<0*.*05 (1-way ANOVA); relative protein levels after stain-free total protein normalization*. *D*: *all values standardised to citrate synthase*. *A*, *B*: *n(CTRL/ELVD/HF) = 4/4/7*. *C*, *D*: *n = 4 for all shown groups*. *CTRL*: *control*. *ELVD*: *early left ventricular dysfunction*. *HF*: *heart failure*. *LM*: *limb muscle*. *TD*: *thoracic diaphragm*. *C I-V*: *ETC complex I-V*. *ETC*: *electron transport chain*.

To scrutinize the molecular alterations in skeletal muscle, an unbiased proteomic screening approach was performed by 2D-DIGE from whole tissue of LM and TD and subsequently from isolated mitochondria of each specimen. The isolation procedure resulted in accumulated and predominantly undestroyed mitochondria as confirmed by western blotting and transmission electron microscopy (**Supplementary material** S1 File **Figure A**). The proteins found to be differentially expressed in progressing disease were mostly allocated to cytosol and mitochondria (Fig 3A). As early as in ELVD, a total of 12 proteins were more expressed compared to CTRL and afterwards identified (Table 1) in LM. 2 of 4 enzymes reaching the predefined fold change ELVD/CTRL (FC) above 2, which is deemed to be of physiologic relevance, were both part of glycolysis (glyceraldehyde-3-phosphate-dehydrogenase FC 2.3x; the rate limiting enzyme pyruvate kinase FC 2.1x). In HF compared to CTRL, the concentrations of 4 cytosolic proteins were altered (Table 2), of which creatine kinase (M-type) reached the FC threshold (FC 2.9x). Additionally, 4 proteins of the cytoskeleton and 2 enzymes of the electron transport chain (ETC) exceeded a FC>2. The picture of up-regulated catabolic enzymes was furthermore equally displayed and confirmed by the results from TD (Tables 3 and 4, Fig 3B). In TD, the proportion of catalytic activity even increased from ELVD to HF.

**Table 1:** Identified proteins comparing ELVD to CTRL in limb muscle.

Identified Protein	NCBI	UniProtID	Orthologue	MOWSE	FC	p-value	Allocation	Function
Creatine kinase M-type	gi|126723370	P00563	CKM	146	1.4	<0.001	Cytosol	Energy transduction
Troponin T, slow skeletal muscle	gi|149016651	Q7TNB2	TNNT1	173	1.6	0.015	Cytoskeleton	Muscle contraction
Pyruvate kinase isozymes M1/M2 isoform 1	gi|307548866	P11974	PKM	241	2.1	0.017	Cytoplasm	Glycolysis
Glyceraldehyde-3-phosphate dehydrogenase	gi|126723533	P46406	GAPDH	270	2.3	0.04	Cytosol	Glycolysis
Myosin light chain 3	gi|291393583	G1T375	MYL3	475	1.9	0.025	Cytosol	Muscle contraction
Aldehyde dehydrogenase 2, mitochondrial	gi|291406975	G1SUY2	ALDH2	386	1.4	0.025	Mitochondrial matrix	Degradation of aldehyde derivatives, ethanol detoxification
Phosphoglucomutase	gi|1942196	P00949	PGM1	84	1.6	0.031	Cytoplasm	Glycogenolysis
Heat shock 27kDa protein 2	gi|291383898	G1T4G1	HSPB2	107	1.2	0.033	Cytoplasm, nucleus	Positive regulation of catalytic activity
Glycerol-3-phosphate dehydrogenase 1	gi|291389121	P08507	GPD1	160	1.5	0.035	Cytoplasm	Glycerol-3-phosphate shuttle
rCG29479, isoform CRA_a	gi|149034378	-*	ZNF709	80	2.3	0.038	Nucleus	Transcriptional regulation
Cytoplasmic beta-actin	gi|291413356	P68135	ACTA1	832	4.6	0.04	Cytoskeleton	Skeletal muscle thin filament assembly
Aconitase 2, mitochondrial	gi|291410318	G1TUX2	ACO2	115	1.9	0.030	Mitochondrial matrix	Tricarboxylic acid cycle

NCBI: NCBI account number. MOWSE: probability-based Molecular Weight SEarch score signifying the significance of protein-identification by peptide mass fingerprint as -10*log_10_(p-value), thus a score>67 corresponds to a p-value<0.05. Orthologue: orthologue genes in rabbits and humans, looked up by the BetterBunny tool v2.3 (11/2015). FC: fold change ELVD/CTRL. p-value: p-value for testing the expression levels ELVD versus CTRL (student’s t-test). ELVD: early left ventricular dysfunction. CTRL: control.

*identification by MALDI-MS/MS in other species than rabbit.

**Table 2:** Identified proteins comparing HF to CTRL in limb muscle.

Identified Protein	NCBI	UniProtID	Orthologue	MOWSE	FC	p-value	Allocation	Function
Myosin-1	gi|296201252	G1TKS9	MYH1	106	2.2	0.022	Myofibril	Muscle contraction
Desmin	gi|284005349	G1SEF9	DES	878	1.8	0.037	Sarcolemma	Muscle contraction
Creatine kinase M-type	gi|126723370	P00563	CKM	589	2.9	0.001	Cytosol	Energy transduction
Glycerol-3-phosphate dehydrogenase 1	gi|291389121	P08507	GPD1	253	1.8	0.024	Cytosol	Glycerol-3-phosphate shuttle
Myosin alkali light chain 3	gi|291393583	G1T375	MYL3	475	1.9	0.027	Cytosol	Muscle contraction
Cytoplasmic beta-actin	gi|291413356	P68135	ACTA1	213	4.5	0.049	Cytoskeleton	Skeletal muscle thin filament assembly
Myosin-4	gi|157954424	Q28641	MYH4	194	2.5	0.047	Myofibril	Microtubule-based movement
ATP synthase subunit d, mitochondrial	gi|291413480	G1T9N2	ATP5PD	90	2.6	0.003	Mitochondrial inner membrane	Part of ATP synthase
Cytochrome b-c1 complex subunit 2, mitochondrial	gi|291390734	P34863	UQCRC2	216	2.0	0.007	Mitochondrial inner membrane	Part of complex III, ETC
ATP synthase subunit alpha, mitochondrial	gi|291394323	G1SKT4	ATP5F1A	699	1.7	0.027	Mitochondrial inner membrane	Part of ATP synthase
Pyruvate kinase PKM isoform 2	gi|307548868	P11974	PKM	115	1.4	0.039	Cytosol	Glycolysis
Cytochrome c oxidase subunit 5A, mitochondrial	gi|655887883	G1TZN7	COX5A	172	1.5	0.045	Mitochondrial inner membrane	Part of complex IV, ETC

NCBI: NCBI account number. MOWSE: probability-based Molecular Weight SEarch score signifying the significance of protein-identification by peptide mass fingerprint as -10*log_10_(p-value), thus a score>67 corresponds to a p-value<0.05. Orthologue: orthologue genes in rabbits and humans, looked up by the BetterBunny tool v2.3 (11/2015). FC: fold change HF/CTRL. p-value: p-value for testing the expression levels HF versus CTRL (student’s t-test). HF: heart failure. CTRL: control. ETC: electron transport chain. ATP: adenosine triphosphate.

**Table 3:** Identified proteins comparing ELVD to CTRL in thoracic diaphragm.

Identified Protein	NCBI	UniProtID	Orthologue	MOWSE	FC	p-value	Allocation	Function
Cytochrome c oxidase subunit 5A, mitochondrial	gi|558121230	.*	COX5A	84	1.3	0.019	Mitochondrial inner membrane	Part of complex IV, ETC
Fatty acid-binding protein	gi|291399415	G1T7R1	FABP3	384	1.7	0.033	Cytoplasm	Intracellular transport of long-chain fatty acids
Cytochrome b-c1 complex subunit Rieske, mitochondrial	gi|655882729	P34863	UQCRFS1	600	2.8	0.012	Mitochondrial inner membrane	Part of complex III, ETC
Glyceraldehyde-3-phosphate dehydrogenase	gi|126723533	P46406	GAPDH	412	3.0	0.018	Cytosol	Glycolysis
Creatine kinase S-type	gi|555985457	.*	CKMT2	246	2.9	0.019	Mitochondrial inner membrane / intermembrane space	Energy transduction
Adenylate kinase 2, mitochondrial isoform X2	gi|655879866	G1SG80	AK2	219	2.3	0.019	Mitochondrial intermembrane space	ADP biosynthetic process

NCBI: NCBI account number. MOWSE: probability-based Molecular Weight SEarch score signifying the significance of protein-identification by peptide mass fingerprint as -10*log_10_(p-value), thus a score>67 corresponds to a p-value<0.05. Orthologue: orthologue genes in rabbits and humans, looked up by the BetterBunny tool v2.3 (11/2015). FC: fold change ELVD/CTRL. p-value: p-value for testing the expression levels ELVD versus CTRL (student’s t-test). ELVD: early left ventricular dysfunction. CTRL: control.

*identification by MALDI-MS/MS in other species than rabbit.

**Table 4:** Identified proteins comparing HF to CTRL in thoracic diaphragm.

Identified Protein	NCBI	UniProtID	Orthologue	MOWSE	FC	p-value	Allocation	Function
Pyruvate Kinase	gi|109157779	P11974	PKM	451	2.0	0.008	Cytosol	Glycolysis
NADH-ubiquinone oxidoreductase 75 kDa subunit, mitochondrial	gi|291392087	G1T359	NDUFS1	459	1.4	0.013	Mitochondrial inner membrane	Part of complex I, ETC
Fructose Bisphosphate Aldolase	gi|160286558	P00883	ALDOA	517	1.3	0.039	Cytoplasm	Glycolysis
Malate dehydrogenase, cytoplasmic	gi|291386712	G1SQG5	MDH1	400	1.5	0.048	Cytoplasm	Tricarboxylic acid cycle

NCBI: NCBI account number. MOWSE: probability-based Molecular Weight SEarch score signifying the significance of protein-identification by peptide mass fingerprint as -10*log_10_(p-value), thus a score>67 corresponds to a p-value<0.05. Orthologue: orthologue genes in rabbits and humans, looked up by the BetterBunny tool v2.3 (11/2015). FC: fold change HF/CTRL. p-value: p-value for testing the expression levels HF versus CTRL (student’s t-test). HF: heart failure. CTRL: control. ETC: electron transport chain.

As integral membrane proteins are difficult to isolate from the lipid bilayer by standard lysis puffer[40] and might therefore be missed by our proteomic approach, we set out to scrutinise their abundance and activity by additional methods: Western Blotting revealed a down-regulated expression of complex IV in LM of the ELVD and HF group (Fig 3C), which did not translate into impaired activity of this complex (Fig 3D). Together, the results of the proteomic screening approach revealed an early shift to catabolism in LM and TD. Particularly, the glycolysis pathway was stressed. Predominantly cytosolic catabolic enzymes were more abundant, whereas ETC complexes remained equally expressed except for complex IV, whose reduced expression level did however not translate into functional relevance. Thus, the cytosolic set-up favouring increased energy production was not matched by corresponding alterations of the mitochondrial proteome. Since pyruvate produced by glycolysis is actively transported across the inner mitochondrial membrane, we wondered, whether the transmembrane transport of metabolites between the cytosol and the mitochondrial matrix could be hit in heart failure induced LM sarcopenia and explain our results.

### Pathway-focused gene expression analysis confirms altered mitochondrial translocation system

As the screening proteomic approach generated the hypothesis of altered mitochondrial transmembrane transport system in tachycardiomyopathy-induced sarcopenia, a gene expression analysis was conducted and focused on mitochondrial transport (GO:0006839), mitochondrial protein import (GO:0030150), protein targeting to mitochondrion (GO:0006626), fatty acid transmembrane transport (GO:1902001) and β-oxidation (GO:0006635). The results comparing HF to CTRL confirmed the hypothesis of an altered mitochondrial transmembrane transport of substrates (Table 5). Transporters of matrix-targeted preproteins showed inhomogeneous results of a less expressed outer membrane transporter (Metaxin2 FC -1.3x) and part of the PAM complex (GrpE FC -1.5x), with slightly more expressed inner membrane translocases (TIMM17 FC 1.2x; TIM 8A FC 1.4x; TIMM10 FC 1.3x). The expression of enzymes facilitating the import of fatty acids into mitochondria was increased (SLC25A20 FC 1.6x; CPT1B FC 2.2x, p<0.05), whereas components of the malate-aspartate shuttle were less expressed in HF (SLC25A12 FC -1.6x; SLC25A13 FC -1.7x; p<0.05). Together, data suggest a transmembrane transport, which favours the supply with fatty acids to intramitochondrial β–oxidation and affects adversely the translocation of reducing equivalents (malate-aspartate shuttle).

**Table 5:** Pathway-focused gene expression analysis comparing HF to CTRL in skeletal muscle.

Gene	FC (HF/CTRL)	Function
Caveolin2	-1.9	Mitogenesis
Fission 1	1.2	Fission
Carnitine palmitoyltransferase 2	2.4	Long chain fatty acid oxidation
Metaxin 2	-1.3	Mitochondrial translocation
GrpE-like 2	-1.5	PAM complex
TIMM17B	1.2	Mitochondrial translocation
Tim 8 A	1.4	Mitochondrial translocation
TIMM 10	1.3	Mitochondrial translocation
SLC25A12	-1.6	Translocation of amino acids
SLC25A13	-1.7	Translocation of amino acids
SLC25A20	1.6	Carnitine shuttle.
CPT1B	2.2	Beta oxidation. Rate limiting step.
MSTO1	-2.2	Fusion/Fission

CTRL: control. HF: heart failure. FC: fold change. PAM-complex: presequence translocase-associated motor (import of preproteins to the mitochondrial matrix). TIMM: mitochondrial import inner membrane translocase. SLC: solute carrier family. CPT1B: carnitine palmitoyltransferase IB. MSTO1: protein misato homolog 1.

### Generating a hypothesis by multi-omics approach—Linking altered enzymatic set-up of LM to PGC-1α pathway and desensitised natriuretic peptide signalling

**Fig 4:**
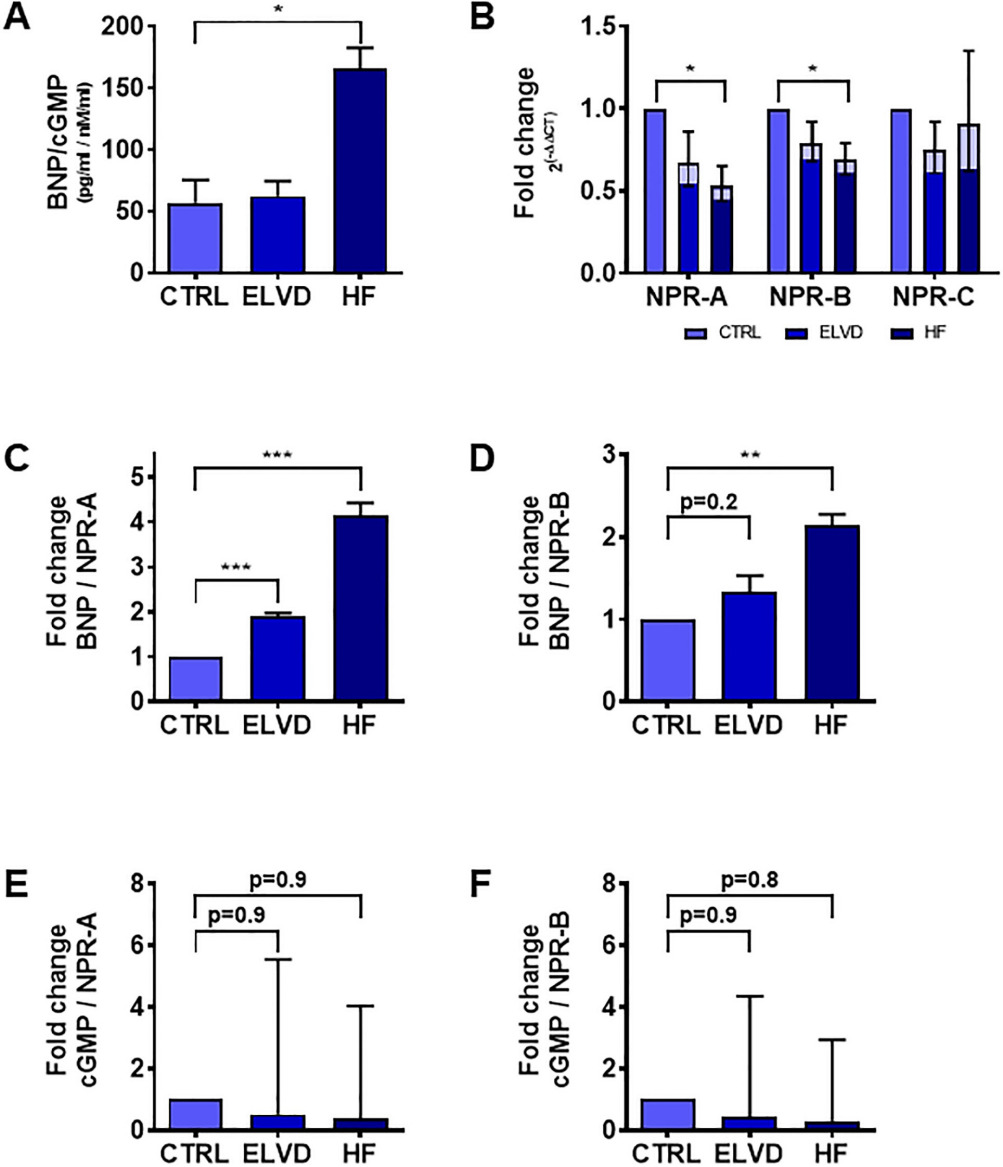
During heart failure progression, skeletal muscle exhibits an attenuated BNP/NPR-A/cGMP signalling and declining expression levels of natriuretic peptide receptors. In heart failure, skeletal muscle was desensitized to BNP as shown by an increased ratio of serum BNP to LM tissue cGMP (**A**). The expression levels of natriuretic peptide receptors A and B in LM decreased with disease progression (**B**). It was accompanied by an increased ratio of serum BNP to NPR-A and B expression in LM (**C, D**), mirroring the extend of reduced BNP/cGMP-quotient (**A**). As the ratio between NPR-A/B and the second messenger cGMP remained stable (**E, F**), the reduced cGMP response to BNP in LM is suggested to be largely due to reduced expression of natriuretic peptide receptors. *A*: n = 3; *p<0.*05 unpaired t-test*. *B*: *n = 4 for each group with the assay run in triplicate*. *All fold changes are referenced to the corresponding CTRL*. *Shown are the geometric means with the according 95%CI*. *p<0.*05 unpaired t-test on the log fold changes (ΔΔCT)*. *C*, *D*: *n = 3/3/2 CTRL/ELVD/HF*. *E*, *F*: *n = 4 for each group*. *C-F*: *Shown are mean and standard deviation*. *All fold changes are referenced to CTRL*. **p<0.*01*, ***p<0.*001 unpaired t-test*. *CTRL*: *control*. *ELVD*: *early left ventricular dysfunction*. *HF*: *heart failure*. *BNP*: *serum B-type natriuretic peptide level*. *cGMP*: *concentration of tissue cyclic guanosine monophosphate*. *NPR-A*, *B*, *C*: *natriuretic peptide receptor A*, *B*, *C*. *LM*: *limb muscle*.

Considering the previous results, the synopsis of muscle-fibre-type shift, boosted glycolytic flux and reduced expression of ETC enzymes suggested suppressed PGC-1α signalling (cf. discussion section), which is under physiologic conditions known to be linked to natriuretic peptide signalling[14]. As in heart failure natriuretic peptide signalling is desensitised[15], we hypothesised, that desensitisation affects similarly LM. Indeed, the ratio of serum BNP to tissue cGMP in LM increased markedly (Fig 4A), whilst natriuretic peptide receptor A (NPR-A) as main receptor of natriuretic peptides was down regulated in ELVD and still more in HF (Fig 4B). The expression of NPR-B was decreased in HF, BNP’s clearance receptor NPR-C was not altered during disease progression. The relationship between BNP and its main receptor NPR-A (Fig 4C) mirrored the attenuated cGMP response (Fig 4A), whereas the ratio of the receptor NPR-A and the second messenger cGMP did not change. Together, resistance to natriuretic peptide signalling in LM of HF was confirmed and might be largely due to decreased expression of its main receptor.

### Combined RAS-/NEP-inhibition prevents loss of protein content in cardiac-induced LM myopathy

**Fig 5:**
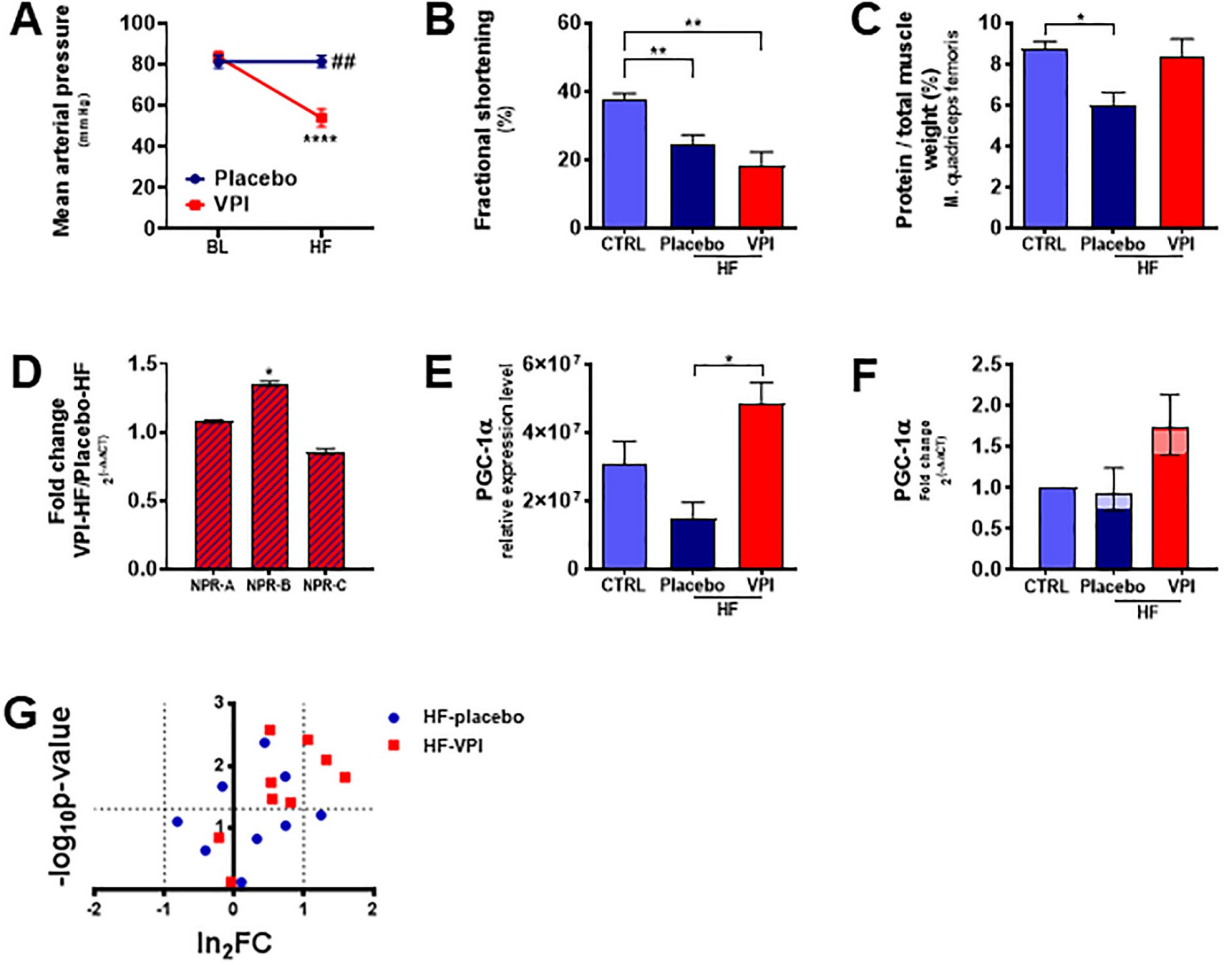
Combined RAS-/NEP-inhibition prevents loss of protein content in skeletal muscle independent of left ventricular function and harmonises the regulation of β-oxidation. The blood pressure lowering effect demonstrated efficacy of combined RAS-/NEP-inhibition in the animal study (**A**). Despite it did not avoid left ventricular systolic dysfunction (**B**), it prevented loss in skeletal muscle protein content (**C**). Natriuretic peptide receptor B was more expressed in VPI treated animals than in the placebo group (both in end-stage heart failure), natriuretic peptide receptor A tended to increase (**D**). A rescue of PGC-1α levels (**E, F**) is supposed to link natriuretic peptide signalling to fatty acid metabolism, as the regulation of key enzymes of β-oxidation steps was harmonised (G). *A*: ****p<0.*0001 Bonferroni post-test for ##p<0*.*01 two-way ANOVA*, *n(Placebo/VPI) = 7/7*. *B*, *E*: *p<0.*05*, **p<0.*01 unpaired t-test*, *n(CTRL/placebo/VPI) = 4/2/2*. *C*: *p<0.*05*, *n = 3 for each group*. *D*: *p<0.*05*, *all fold changes are referenced to placebo*. *Shown are the geometric means with the according 95%CI*. *n(Placebo/VPI) = 7/7*. *E*: *relative protein levels after stain-free total protein normalization*. *F*: *all fold changes are referenced to CTRL*. *Shown are the geometric means with the according 95%CI*. *G*: *volcano plot*, *enzymes of* β-oxidation (GO pathway 436.659, 436.660, 436.661), n = 4 animals for each group. *n(CTRL/placebo/VPI) = 5/7/7*. *RAS-/NEP-inhibition*: *combined inhibition of the renin-angiotensin-aldosterone system and neprilysin (omapatrilat)*. *BL*: *baseline*. *CTRL*: *control*. *HF*: *heart failure*. *VPI*: *omapatrilat*, *combined RAS/NEP-inhibitor*. *NPR-A*, *B*, *C*: *natriuretic peptide receptor A*, *B*, *C*. *PGC-1*α: peroxisome-proliferator-activated-receptor-γ-coactivator-1-α.

In consequence, we hypothesised that a loss in LM protein content as surrogate of cachexia could be prevented by combined RAS-/NEP-inhibition. Similar to the descriptive animal study, congestive heart failure was again provoked by incremental tachypacing during 30 days. Seven animals were treated by a combined RAS-/NEP-inhibition, seven got placebo during the pacing period. Treatment lowered the mean arterial pressure by 33.7%, showing efficacy of combined RAS-/NEP-inhibition (Fig 5A). Left ventricular systolic dysfunction was established in both groups (Fig 5B). Though fractional shortening did not differ between treatment and placebo group, LM protein content was preserved by combined RAS-/NEP-inhibition in marked contrast to placebo (Fig 5C). It confirmed the hypothesis of the pharmacological intervention study. NPR-B was more expressed in LM of HF-VPI animals than in the HF-placebo group, NPR-A tended to increase (Fig 5D). Furthermore, RAS-/NEP-inhibition tends to rescue the expression levels of PGC-1α (Fig 5E and 5F). The regulation of key enzymes of β-oxidation steps was harmonised (Fig 5G), matching the assumption of mediation by PGC-1α.

## Discussion

Our study reports for the first time, that the animal model of tachypacing-induced heart failure entails a generalized myopathy, occurring very early—even preceding a deterioration of systolic function. The progression from an early metabolic remodelling towards structural alterations of skeletal muscle mirrors aspects of human disease[41]. In respect of previous animal models of cardiac-induced myopathy[8,42–44], our model offers the unique possibility of characterising the “pre-cachectic” state[7] and the timely dimension of disease. The multi-omics approach confirmed previous data, provided new insights into the enzymatic remodelling underlying futile substrate metabolism and led to a new hypothesis, linking heart failure induced desensitisation of natriuretic peptide signalling to skeletal muscle catabolism. Concurrently, an interventional study validated a beneficial effect of combined RAS-/NEP-inhibition on cardiac-induced myopathy.

### Animal model—Translational implications

The typical human entity of tachycardia-induced heart failure can be fully resolved after cessation of the causal arrhythmia[45]. Diagnosis is often complicated by the reciprocal causal link between arrhythmia and left ventricular dysfunction, as most structural heart diseases can lead to arrythmia themselves[46]. In typical forms, effective interventional treatments of e.g. atrial fibrillation[47] have been developed and proved clinical benefit in heart failure[48]. The enduring complete reversibility is a rare characteristic beneath the other aetiologies of heart failure syndrome[2], as coronary artery disease and most structural cardiomyopathies. They induce cardiac remodelling and lead to a vicious cycle, that is further driven by excessive neurohumoral stimulation[49]. Symptomatic heart failure affects more than 8% in the elderly [50,51]. Despite optimal treatment recommended by the current guidelines[2], mortality is reported about 17% during a follow-up period of 27 months in recent pharmacological studies [52]. Albeit tachypacing-induced heart failure mimics an aetiology being nowadays curable in humans, the tachypacing-induced heart failure model represents accurately the progressive nature of heart failure[9,10,12] as well as the final common neurohumoral pathway of the highly prevalent human heart failure syndrome[12,53–55]. These particular features qualify especially this model for our current research purpose: other surgical procedures (e.g., ligation of coronary arteries[44], transverse aortic constriction[42]) do not display a chronic and gradual progression of heart failure induced myopathy as recently reviewed[8], albeit the assessment of early alterations could indicate new diagnostic or even therapeutic aspects for prevention[41].

Our animal model mirrored some hallmarks of the human disease state: as in heart failure patients, the fibre type distribution was altered with an augmented percentage of fast twitch fibres[56]. The enzymatic set-up in LM and TD resembles metabolic shift to anaerobic glycolysis, which can be seen in limb muscles of human heart failure patients by ^31^P magnetic resonance spectroscopy[57].

An unsettled issue in human heart failure is the differentiation between effects of inactivity and systemic metabolic remodelling on skeletal muscle. In chronic, systemic diseases, patients tend to avoid physical exercise and inactivity causes skeletal muscle alterations itself[58]. The proteomic signature of inactive skeletal muscle in humans at long term bed rest has been recently reviewed[59]. It comprises a down-regulation of enzymes belonging to oxidative metabolism. A study using 2-D DIGE in muscle biopsies of patients at long term bed rest reports particularly decreased isoforms of aconitase[60]. This particular finding on the level of the single enzyme aconitase as well as the whole picture of the down regulated pathway of oxidative metabolism contrasts our results of elevated catabolism with increased glycolytic flux and up-regulated aconitase. Thus, the proteome profile in our study can be set apart from published proteomic signatures of atrophy. To a greater degree, our results resemble aspects of cardiac-induced myopathy in humans, which is frequently characterised by early catabolic dominance, particularly boosted glycolysis[41], preceding loss in function[61].

To further scrutinise the role of inactivity in our model, TD was additionally evaluated. Albeit TD is in life-long, constant use[62], heart failure patients suffer from a loss in respiratory muscle strength[63], which increases dead-space ventilation and aggravates ventilation-perfusion mismatch during exercise[64]. Our animal model showed accordingly a generalised myopathy of LM and TD. Proteomics revealed a similar catabolic dominance in the diaphragm.

Together, our model mimics aspects of skeletal muscle alterations in human heart failure. Even if inactivity may have an additional effect on skeletal muscle in our model, the metabolic remodelling of skeletal muscle seems to be induced by systemic alterations. In the translational context, our animal model might provide additional value in respect of existing models, since it allows for the experimental evaluation of early, systemic induced skeletal muscle remodelling as well as of the progression from myopathy to heart failure provoked cachexia.

### Remodelling of metabolic enzymes in heart failure induced sarcopenia—Catabolic dominance and futile cycles

From a metabolic view, heart failure induced sarcopenia and cachexia is largely recognised as state of catabolic dominance[65]: this is well mirrored by the increased expression or abundance of enzymes catalysing glycolysis and β-oxidation in our HF group, which we could already describe in an very early stage of heart failure development (ELVD) in line with recent literature[41]. Beyond this broad term of catabolism, the transcriptomic and proteomic screening substantiates the understanding of underlying enzymatic remodelling: the increased abundance of pyruvate kinase can facilitate an augmented glycolytic flux, entailing an increased cytosolic consumption of NAD^+^ to produce NADH. As the inner mitochondrial membrane is impermeable to NADH/NAD^+^[66], NADH depends on the malate-aspartate shuttle, that ensures the transmembrane transport of electrons to serve further energy production (oxidative phosphorylation) and to replenish the cytosolic NAD^+^ pool for glycolysis[67]. Core enzymes of this shuttle were found decreased in our HF group. It is tempting to speculate, that the coincidence of decreased shuttling capacity and increased glycolytic enzymes favours the regeneration of NADH by lactate dehydrogenase resulting in increased lactate levels, not necessarily associated with hypoxia[68]. Consistently, lactate dehydrogenase activity is increased in quadriceps of a heart failure model using ligation of the left anterior descending coronary artery in rats[44]. In humans, exercise magnetic resonance spectroscopy of the flexor digitorum superficialis muscle reported a lower intracellular pH at each workload in heart failure patients compared to healthy volunteers[69]. Consistently, our measurements showed an increase in serum lactate concentrations. Elevated blood lactate levels have been known in heart failure patients since 1958[70]. Despite marked progress in treatment regimens[2,71], elevated lactate is still part of the metabolic fingerprint of heart failure[72] and common in patients admitted to hospital for heart failure[73].

Additionally, the revealed enzymatic remodelling might further aggravate futile substrate metabolism as described for cancer cachexia[74]: As the proton of NAD^+^ + H^+^ contributes to the proton-motive force in the mitochondrial intermembrane space and pyruvate delivers more energy by means of ATP by being introduced to citric acid cycle and oxidative phosphorylation, the conversion of glucose into lactic acid is an energy-inefficient process[75]. LM producing lactate resembles much to the accelerated Cori cycle in cancer cachexia[76], a futile cycle increasing unnecessary energy consumption: lactate produced by the tumour is regenerated to glucose by the liver consuming ATP. The regenerated glucose is recycled to the tumour, which breaks it down to lactate again[77]. Together, our unbiased, hypothesis-free screening approach generated a novel hypothesis, which our results and published data would congruously fit and which is subjected to further experimental assessment.

### PGC-1α and natriuretic peptides—Combined inhibition of RAS/NEP ameliorates heart failure induced cachexia

Forming an overall perspective of the results so far, the histological and metabolic findings drew the picture of an suppressed PGC-1α signalling, as PGC-1α exerts pleiotropic effects[78], which are opposite to our findings: PGC-1α mediates the maintenance of normal muscle fibre-type composition[79,80], suppresses the glycolytic flux[81] and increases the expression of genes involved in oxidative phosphorylation[82]. Of particular note, PGC-1α expression is linked to natriuretic peptides, pivotal cardiac hormones involved in heart failure disease[12,83]. Activation of NPR-A induces PGC-1α gene expression in a cGMP-dependent manner in human myotubes[14]. Interestingly, with progression of heart failure the functional effectiveness of natriuretic peptides becomes blunted as recently reviewed[15]. The ratio of cGMP to BNP is an adequate index of the effectiveness of BNP [15,84–87]. In our study, LM showed a diminished responsiveness to BNP. Additionally, NPR-A-expression was decreased in LM during disease progression. Apart from reduced NPR-A expression [88], other mechanisms are known to diminish target organ responsiveness as receptor desensitization[89,90] and inhibited downstream signalling[91]. To get an impression of the effect size of the measured NPR-A-downregulation on the attenuation of the BNP/NPR-A/cGMP pathway in heart-failure induced skeletal muscle remodelling the quotients serum BNP to NPR-A expression as well as tissue cGMP to NPR-A were computed. The ratio of the active hormone BNP to its main receptor increased remarkably (Fig 4C) and mirrored the extend of pathway attenuation (Fig 4A), suggesting a relevant effect of the observed receptor-downregulation. As the ratio between cGMP and NPR-A remained unchanged, a relevant role of NPR-A desensitization or inhibited downstream signalling as alternative explanation for blunted BNP/NPR-A/cGMP-signalling in heart-failure induced skeletal muscle remodelling seems rather unlikely.

In conspectus of (I) the attenuated BNP/NPR-A/cGMP pathway in LM of HF animals, (II) the published link between natriuretic peptide signalling and PGC-1α expression and (III) histological, transcriptomic and proteomic hallmarks of reduced PGC-1α activity, we hypothesised that a pharmacological intervention, increasing the availability of biological active BNP without aggravating effector desensitization[15], increases PGC-1α expression and thus, ameliorates protein content of skeletal muscle as surrogate marker of catabolic dominance and clinically, cachexia. For evaluating the hypothesis, an interventional study was designed, which compared in the animal model of tachypacing-induced heart failure a group with symptomatic heart failure treated with omapatrilat during 30days of pacing to a group fed with placebo. Omapatrilat is the leading substance of vasopeptidase inhibitors (VPI)[92], that inhibit the angiotensin converting enzyme as well as the natriuretic peptides degrading enzyme neprilysin[12,93] and paved the way for angiotensin-receptor neprilysin-inhibitors[52]. VPI increased PGC-1α-levels in LM of heart failure animals and prevented LM from protein loss, despite no effect of VPI on left ventricular systolic function was seen in our sample. Summarising, based on published literature and our transcriptomic and proteomic screening approach, our data provide evidence for a hitherto unrecognised beneficial effect of combined RAS/NEP-inhibition on heart-failure induced cachexia, which further broadens the favourable effect spectrum of NEP-inhibition in heart failure. It is tempting to speculate about substantial clinical relevance, as mortality and the loss in quality of life due to muscle weakness and cachexia are frequent and still coming to the fore[41] and a combination drug comprising NEP-inhibition has recently been approved, marketed and recommended by current guidelines[2,52]. It ought to be subjected to further clinical studies, whether the beneficial effect of NEP-inhibition on skeletal muscle is transferrable to humans. Beyond pharmacological management, the results might entail a further treatment option: PGC-1α is targetable by long-term physical activity[94,95] and could additionally explain advantageous impacts of physical exercise on heart failure outcome[96,97].

### Limitations

Some limitations have to be carefully respected: the ratio of serum BNP to tissue cGMP is commonly deemed to represent the effectiveness of natriuretic peptide signalling[15,84–87,98]. Thus, BNP application elevates cGMP levels in experimental heart failure[87]. However, tissue cGMP is also produced coupled to nitric oxide signalling or even independent of natriuretic peptides and nitric oxide[99]. Together, it cannot be ruled out, that other signalling pathways influence the BNP/cGMP-ratio. But aside from the BNP/cGMP-ratio, out initial suggestion of desensitised natriuretic peptide signalling is additionally strengthened by elevated BNP and decreased NPR-A. Together with all necessary caution in interpretation, the elevated ratio is a congruent finding, fitting the altered natriuretic peptide signalling axis.

Concerning the treatment study, omapatrilat was given instead of the novel combination drug sacubitril/valsartan[52]. Unfortunately, at the time we designed the trial and applied for permission of the governmental animal care committee, sacubitril/valsartan was not available. Due to very similar modes of action of NEP inhibition[100], a class effect appears likely, but has to be validated in further studies.

Omapatrilat combines the inhibition of two modes of action: inhibition of ACE and of NEP[92]. Hence, we cannot rule out a potential beneficial effect on skeletal muscles by ACE-inhibition with additive value to NEP-inhibition or even outperforming the NEP-inhibition. However, since NEP inhibition results necessarily in vivo in RAS-activation, NEP inhibition requires unavoidably concomitant RAS-inhibition to be of any clinical benefit[101,102] and a sole NEP inhibition in an in vivo study did not appear reasonable. Notwithstanding, published work proved a mechanistic link between natriuretic peptides and PGC-1α in vitro[14], that provided the basis of our in-vivo experiments. The beneficial effect was additionally independent of left ventricular ejection fraction.

Neprilysin degrades further substrates apart from natriuretic peptides, e.g. oxytocin, gastrin endothelin-1 and several others[103]. Thus, alternative effect pathways between VPI and mitigated protein loss than BNP/NPR-A/PGC-1α cannot be ruled out. With respect to these limitations, our data cannot actually prove causality of reinforced BNP/NPR-A/PGC-1α-pathway for ameliorated myopathy in heart failure, but they may contribute to the growing evidence for this mechanistic path as discussed above[14].

### Conclusion

Tachypacing-induced heart failure entails a generalised myopathy, preceding deterioration of systolic function. By mirroring the time perspective of cardiac-induced myopathy in humans, the model provides a unique feature. The early hypercatabolic state of skeletal muscles comprises enzymatic remodelling, which renders the tissue prone to futile substrate metabolism. A combined RAS-/NEP-inhibition ameliorates cardiac-induced myopathy independent of systolic function, which could be linked to stabilised NP/cGMP/ PGC-1α signalling.

## Supporting information

S1 File **Table A: Gene lists for pathway-focused gene expression analysis: fatty acid metabolism. Table B: Gene lists for pathway-focused gene expression analysis: Mitochondria. Table C: Gene lists for pathway-focused gene expression analysis: PPAR Targets. Figure A: Mitochondria were sufficiently isolated from whole LM tissue**. (A) After isolation of mitochondria, detecting specific proteins of mitochondrial matrix (Hsp60), cytosol (β-actin), outer (VDAC) and inner (cytochrome c) mitochondrial membrane by western blot validated the enriched number of mitochondria. (B) The isolated mitochondria were in large majority undestroyed (aside from matrix oedema related to sample processing), as visualised by transmission electron microscopy. *Representative images of western blot (A) and transmission electron microscopy (B)*. *LM*: *limb muscle*. *CTRL*: *control animal*. *PC*: *positive control (lysates of whole LM tissue from rats)*. *ELVD*: *early left ventricular dysfunction*.
(DOCX)
