# Supplementary material for: Skeletal muscle alterations in tachycardia-induced heart failure are linked to deficient natriuretic peptide signalling and are attenuated by RAS-/NEP-inhibition
Source: PLoS One. 2019 Dec 4;14(12):e0225937. doi: 10.1371/journal.pone.0225937 (PMC6892497; doi:10.1371/journal.pone.0225937)
Supplement: S1 File — Table A: Gene lists for pathway-focused gene expression analysis: fatty acid metabolism. Table B: Gene lists for pathway-focused gene expression analysis: Mitochondria. Table C: Gene lists for pathway-focused gene expression analysis: PPAR Targets. Figure A: Mitochondria were sufficiently isolated from whole LM tissue. (A) After isolation of mitochondria, detecting specific proteins of mitochondrial matrix (Hsp60), cytosol (β-actin), outer (VDAC) and inner (cytochrome c) mitochondrial membrane by western blot validated the enriched number of mitochondria. (B) The isolated mitochondria were in large majority undestroyed (aside from matrix oedema related to sample processing), as visualised by transmission electron microscopy. Representative images of western blot (A) and transmission electron microscopy (B). LM: limb muscle. CTRL: control animal. PC: positive control (lysates of whole LM tissue from rats). ELVD: early left ventricular dysfunction. (DOCX) [file pone.0225937.s001.docx]

**S1 File Table A: Gene lists for pathway-focused gene expression analysis:**

**fatty acid metabolism**

.

| **NCBI Gene ID** | **GenBank** | **Symbol** | **Description** |
| --- | --- | --- | --- |
| 100340554 | XM_008252713 | ACAA1 | Acetyl-CoA acyltransferase 1 |
| 100339733 | XM_002713530 | ACAA2 | Acetyl-CoA acyltransferase 2 |
| 100338272 | XM_002719769 | ACAD10 | Acyl-CoA dehydrogenase family, member 10 |
| 100346240 | XM_008248361 | ACAD8 | Acyl-CoA dehydrogenase family, member 8 |
| 100343898 | XM_002713167 | ACAD9 | Acyl-CoA dehydrogenase family, member 9 |
| 100358521 | XM_002712562 | ACADL | Acyl-CoA dehydrogenase, long chain |
| 100347504 | XM_002715911 | ACADM | Acyl-CoA dehydrogenase, C-4 to C-12 straight chain |
| 100352290 | XM_002719813 | ACADS | Acyl-CoA dehydrogenase, C-2 to C-3 short chain |
| 100351868 | XM_002722594 | ACADSB | Acyl-CoA dehydrogenase, short/branched chain |
| 100346654 | XM_008270808 | ACADVL | Acyl-CoA dehydrogenase, very long chain |
| 100009537 | XM_002715038 | ACAT1 | Acetyl-CoA acetyltransferase 1 |
| 100340500 | XM_002714983 | ACAT2 | Acetyl-CoA acetyltransferase 2 |
| 100344258 | XM_002719663 | ACOT1 | Acyl-CoA thioesterase 1 |
| 100338318 | XM_002713909 | ACOT12 | Acyl-CoA thioesterase 12 |
| 100342993 | XM_008272071 | ACOT6 | Acyl-CoA thioesterase 6 |
| 100354701 | XM_002716151 | ACOT7 | Acyl-CoA thioesterase 7 |
| 100338853 | XM_002721235 | ACOT8 | Acyl-CoA thioesterase 8 |
| 100343088 | XM_002719964 | ACOT9 | Acyl-CoA thioesterase 9 |
| 100338450 | XM_002722933 | ACOX1 | Acyl-CoA oxidase 1, palmitoyl |
| 100009549 | NM_001082763 | ACOX2 | Acyl-CoA oxidase 2, branched chain |
| 103347455 | XM_008253979 | ACOX3 | Acyl-CoA oxidase 3, pristanoyl |
| 100338354 | XM_002721592 | ACSBG1 | Acyl-CoA synthetase bubblegum family member 1 |
| 100350310 | XM_002723973 | ACSBG2 | Acyl-CoA synthetase bubblegum family member 2 |
| 100340861 | XM_002719291 | ACSF2 | Acyl-CoA synthetase family member 2 |
| 100358351 | XM_002712500 | ACSL3 | Acyl-CoA synthetase long-chain family member 3 |
| 100342485 | XM_002720191 | ACSL4 | Acyl-CoA synthetase long-chain family member 4 |
| 100344347 | XM_008270510 | ACSL5 | Acyl-CoA synthetase long-chain family member 5 |
| 100328719 | NM_001171343 | ACSL6 | Acyl-CoA synthetase long-chain family member 6 |
| 100345414 | XM_002711809 | ACSM3 | Acyl-CoA synthetase medium-chain family member 3 |
| 100350992 | XM_002711831 | ACSM4 | Acyl-CoA synthetase medium-chain family member 4 |
| 100352493 | XM_002711766 | ACSM5 | Acyl-CoA synthetase medium-chain family member 5 |
| 100338523 | XM_002719770 | ALDH2 | Aldehyde dehydrogenase 2 family (mitochondrial) |
| 100339007 | XM_002716523 | BDH1 | 3-hydroxybutyrate dehydrogenase, type 1 |
| 100344675 | XM_002717204 | BDH2 | 3-hydroxybutyrate dehydrogenase, type 2 |
| 100353138 | XM_002723234 | CPT1B | Carnitine palmitoyltransferase 1B (muscle) |
| 100346210 | XM_002715617 | CPT2 | Carnitine palmitoyltransferase 2 |
| 100344633 | XM_008252728 | CRAT | Carnitine O-acetyltransferase |
| 100355549 | XM_002713837 | CROT | Carnitine O-octanoyltransferase |
| 100352241 | XM_002710572 | DECR1 | 2,4-dienoyl CoA reductase 1, mitochondrial |
| 103347237 | XM_008253719 | DECR2 | 2,4-dienoyl CoA reductase 2, peroxisomal |
| 100341040 | XM_008270644 | ECHS1 | Enoyl CoA hydratase, short chain, 1, mitochondrial |
| 100348349 | XM_002720949 | ECI2 | Enoyl-CoA delta isomerase 2 |
| 100344001 | XM_002718575 | ELOVL3 | ELOVL fatty acid elongase 3 |
| 100353429 | XM_002714545 | ELOVL4 | ELOVL fatty acid elongase 4 |
| 100354672 | XM_002709637 | FABP1 | Fatty acid binding protein 1, liver |
| 100354372 | XM_002717226 | FABP2 | Fatty acid binding protein 2, intestinal |
| 100348000 | XM_002716060 | FABP3 | Fatty acid binding protein 3, muscle and heart (mammary-derived growth inhibitor) |
| 100009416 | XM_002710655 | FABP4 | Fatty acid binding protein 4, adipocyte |
| 100358103 | XM_002714798 | FABP7 | Fatty acid binding protein 7, brain |
| 100342403 | XM_008274405 | FADS1 | Fatty acid desaturase 1 |
| 100352712 | XM_002721017 | FADS2 | Fatty acid desaturase 2 |
| 100343376 | XM_002724235 | GCDH | Glutaryl-CoA dehydrogenase |
| 100354726 | XM_008272484 | GK | Glycerol kinase |
| 100344828 | XM_002717093 | GK2 | Glycerol kinase 2 |
| 100358543 | XM_008266275 | GK5 | Glycerol kinase 5 (putative) |
| 100339469 | XM_008256441 | GPD1 | Glycerol-3-phosphate dehydrogenase 1 (soluble) |
| 100101571 | XM_002712189 | GPD2 | Glycerol-3-phosphate dehydrogenase 2 (mitochondrial) |
| 100355022 | XM_002709814 | HADHA | Hydroxyacyl-CoA dehydrogenase/3-ketoacyl-CoA thiolase/enoyl-CoA hydratase (trifunctional protein), alpha subunit |
| 100344837 | XM_002715980 | HMGCL | 3-hydroxymethyl-3-methylglutaryl-CoA lyase |
| 100338744 | XM_002714013 | HMGCS1 | 3-hydroxy-3-methylglutaryl-CoA synthase 1 (soluble) |
| 100343479 | XM_002715673 | HMGCS2 | 3-hydroxy-3-methylglutaryl-CoA synthase 2 (mitochondrial) |
| 100339941 | XM_008249691 | LIPE | Lipase, hormone-sensitive |
| 100340171 | NM_001177330 | LPL | Lipoprotein lipase |
| 100339203 | XM_002723646 | MCAT | Malonyl CoA:ACP acyltransferase (mitochondrial) |
| 100343924 | XM_002718291 | MCEE | Methylmalonyl CoA epimerase |
| 100349933 | XM_002716066 | MECR | Mitochondrial trans-2-enoyl-CoA reductase |
| 100357233 | XM_002714489 | MUT | Methylmalonyl CoA mutase |
| 100356813 | XM_002714003 | OXCT1 | 3-oxoacid CoA transferase 1 |
| 100343648 | XM_002712578 | PECR | Peroxisomal trans-2-enoyl-CoA reductase |
| 100340178 | XM_008270012 | PPA1 | Pyrophosphatase (inorganic) 1 |
| 100358166 | XM_008262151 | PRKAA1 | Protein kinase, AMP-activated, alpha 1 catalytic subunit |
| 100340670 | XM_008265196 | PRKAA2 | Protein kinase, AMP-activated, alpha 2 catalytic subunit |
| 100348780 | XM_002719800 | PRKAB1 | Protein kinase, AMP-activated, beta 1 non-catalytic subunit |
| 100340412 | XM_002715664 | PRKAB2 | Protein kinase, AMP-activated, beta 2 non-catalytic subunit |
| 100008815 | NM_001082109 | PRKACA | Protein kinase, cAMP-dependent, catalytic, alpha |
| 100342212 | XM_002715892 | PRKACB | Protein kinase, cAMP-dependent, catalytic, beta |
| 100356542 | XM_002711089 | PRKAG1 | Protein kinase, AMP-activated, gamma 1 non-catalytic subunit |
| 100353180 | XM_008263910 | PRKAG2 | Protein kinase, AMP-activated, gamma 2 non-catalytic subunit |
| 100352581 | XM_002712482 | PRKAG3 | Protein kinase, AMP-activated, gamma 3 non-catalytic subunit |
| 100352860 | XM_002717806 | SLC27A2 | Solute carrier family 27 (fatty acid transporter), member 2 |
| 100340848 | XM_002715490 | SLC27A3 | Solute carrier family 27 (fatty acid transporter), member 3 |
| 100352217 | XM_002722970 | SLC27A4 | Solute carrier family 27 (fatty acid transporter), member 4 |
| 100340287 | XM_002721897 | SLC27A5 | Solute carrier family 27 (fatty acid transporter), member 5 |
| 100349387 | XM_002710184 | SLC27A6 | Solute carrier family 27 (fatty acid transporter), member 6 |

**S1 File Table B: Gene lists for pathway-focused gene expression analysis:**

**mitochondria**

| **NCBI Gene ID** | **GenBank** | **Symbol** | **Description** |
| --- | --- | --- | --- |
| 100343674 | XM_002718439 | AIFM2 | Apoptosis-inducing factor, mitochondrion-associated, 2 |
| 100345791 | XM_002719726 | AIFM3 | Apoptosis-inducing factor, mitochondrion-associated, 3 |
| 100347082 | XM_002718845 | LOC100347082 | Aryl hydrocarbon receptor interacting protein-like 1 |
| 100353706 | XM_002717648 | AKT3 | V-akt murine thymoma viral oncogene homolog 3 (protein kinase B, gamma) |
| 100342046 | XM_002714580 | LOC100342046 | BCL2-antagonist/killer 1-like |
| 100008875 | NM_001082135 | BCL2L1 | BCL2-like 1 |
| 100355991 | XM_002722547 | LOC100355991 | BH3 interacting domain death agonist-like |
| 100348536 | XM_002723787 | LOC100348536 | BCL2/adenovirus E1B 19kD-interacting protein 3-like |
| 100126563 | NM_001171022 | CAV2 | Caveolin 2 |
| 100340625 | XM_002707963 | LOC100340625 | Cyclin-dependent kinase inhibitor 2A |
| 100356833 | XM_002718961 | LOC100356833 | COX10 homolog, cytochrome c oxidase assembly protein, heme A: farnesyltransferase-like |
| 100355645 | XM_002718611 | LOC100355645 | Cytochrome oxidase assembly factor-like |
| 100345540 | XM_002721675 | LOC100345540 | COX16 cytochrome c oxidase assembly homolog |
| 100355311 | XM_002717114 | LOC100355311 | Mitochondrial COX18 |
| 100353138 | XM_002723234 | LOC100353138 | Carnitine palmitoyltransferase 1B |
| 100346210 | XM_002715617 | CPT2 | Carnitine palmitoyltransferase 2 |
| 100348759 | XM_002716338 | LOC100348759 | DnaJ homolog, subfamily C, member 19 |
| 100338814 | XM_002712679 | LOC100338814 | Dynamin 1-like |
| 100341062 | XM_002722836 | LOC100341062 | Tetratricopeptide repeat domain 11-like |
| 100342383 | XM_002714508 | GCLC | Glutamate-cysteine ligase, catalytic subunit |
| 100352174 | XM_002715852 | LOC100352174 | Glutamate cysteine ligase, modifier subunit-like |
| 100009258 | NM_001085444 | GPX1 | Glutathione peroxidase 1 |
| 100341336 | XM_002710299 | LOC100341336 | GrpE-like 1, mitochondrial-like |
| 100338694 | XM_002721722 | LOC100338694 | Heat shock 90kDa protein 1, beta |
| 100349912 | XM_002712368 | HSPD1 | Heat shock 60kDa protein 1 (chaperonin) |
| 100358499 | XM_002709031 | LOC100358499 | Uncharacterized LOC100358499 |
| 100337704 | XM_002709601 | IMMT | Inner membrane protein, mitochondrial |
| 100357043 | XM_002709900 | LOC100357043 | Leucine-rich PPR motif-containing protein |
| 100346474 | XM_002716333 | LOC100346474 | Mitofusin 1 |
| 100355371 | XM_002712797 | MIPEP | Mitochondrial intermediate peptidase |
| 100349987 | XM_002709955 | LOC100349987 | Mpv17 protein |
| 100352018 | XM_002715413 | MSTO1 | Misato homolog 1 (Drosophila) |
| 0 | XR_085162 | LOC100357146 | Metaxin 1 |
| 100337978 | XM_002712209 | LOC100337978 | Metaxin 2-like |
| 0 | XR_084882 | LOC100353082 | Neurofilament, light polypeptide 68kDa |
| 100344494 | XM_002716386 | OPA1 | Optic atrophy 1 (autosomal dominant) |
| 100340909 | XM_002712024 | LOC100340909 | Mitochondrial processing peptidase beta subunit |
| 100349219 | XM_002709377 | PPARGC1A | Peroxisome proliferator-activated receptor gamma, coactivator 1 alpha |
| 0 | XR_085313 | LOC100351349 | Ras homolog gene family, member T1 |
| 100353622 | XM_002718790 | LOC100353622 | Ring finger protein 135-like |
| 100358179 | XM_002716099 | LOC100358179 | Stratifin-like |
| 100352421 | XM_002715522 | LOC100352421 | SH3-containing protein SH3GLB1 |
| 100351112 | XM_002722592 | LOC100351112 | Solute carrier family 25, member 1 |
| 100348722 | XM_002724430 | LOC100348722 | Solute carrier family 25 (mitochondrial carrier; dicarboxylate transporter), member 10-like |
| 100350328 | XM_002712180 | LOC100350328 | Solute carrier family 25, member 12-like |
| 100351328 | XM_002713690 | LOC100351328 | Solute carrier family 25, member 13-like |
| 100348432 | XM_002720292 | SLC25A14 | Solute carrier family 25 (mitochondrial carrier, brain), member 14 |
| 100356921 | XM_002721098 | LOC100356921 | Mitochondrial ornithine transporter 1 |
| 100347684 | XM_002718453 | LOC100347684 | Solute carrier family 25, member 16 |
| 100358473 | XM_002721410 | LOC100358473 | Solute carrier family 25 (mitochondrial carrier; peroxisomal membrane protein, 34kDa), member 17 |
| 100342929 | XM_002722941 | LOC100342929 | Mitochondrial thiamine pyrophosphate carrier-like |
| 100351232 | XM_002710131 | LOC100351232 | Solute carrier family 25 member 2-like |
| 100352079 | XM_002713398 | LOC100352079 | Carnitine/acylcarnitine translocase |
| 100009567 | NM_001082777 | SLC25A24 | Solute carrier family 25 (mitochondrial carrier; phosphate carrier), member 23 |
| 100353487 | XM_002722974 | SLC25A25 | Solute carrier family 25 (mitochondrial carrier; phosphate carrier), member 25 |
| 100347746 | XM_002714467 | SLC25A27 | Solute carrier family 25, member 27 |
| 100359028 | XM_002711214 | LOC100359028 | Solute carrier family 25 member 3 |
| 100338986 | XM_002712959 | LOC100338986 | Solute carrier family 25, member 30 |
| 100346477 | XM_002717262 | LOC100346477 | Solute carrier family 25 (mitochondrial carrier; adenine nucleotide translocator), member 31 |
| 100009414 | NM_001082686 | SLC25A4 | Solute carrier family 25 (mitochondrial carrier; adenine nucleotide translocator), member 4 |
| 100009313 | NM_001082627 | SOD1 | Superoxide dismutase 1, soluble |
| 100354978 | XM_002719341 | STARD3 | StAR-related lipid transfer (START) domain containing 3 |
| 100328759 | NM_001171376 | TAZ | Tafazzin |
| 100349306 | XM_002709160 | LOC100349306 | Translocase of inner mitochondrial membrane 10 homolog |
| 100355100 | XM_002708763 | LOC100355100 | Fractured callus expressed transcript 1-like |
| 100358025 | XM_002717598 | LOC100358025 | Translocase of inner mitochondrial membrane 17 homolog A |
| 0 | XR_085360 | LOC100345112 | Translocase of inner mitochondrial membrane 17 homolog B |
| 100338681 | XM_002718896 | LOC100338681 | Translocase of inner mitochondrial membrane 22 homolog |
| 100341303 | XM_002720066 | LOC100341303 | Translocase of inner mitochondrial membrane 23 (yeast) homolog |
| 100353480 | XM_002722003 | LOC100353480 | Translocase of inner mitochondrial membrane 44 |
| 100356545 | XM_002711448 | LOC100356545 | Translocase of inner mitochondrial membrane 50 homolog |
| 100348662 | XM_002715995 | LOC100348662 | Translocase of inner mitochondrial membrane 8 homolog A-like |
| 100346423 | XM_002708437 | LOC100346423 | Translocase of inner mitochondrial membrane 8 homolog b-like |
| 100338928 | XM_002718273 | LOC100338928 | Translocase of inner mitochondrial membrane 9 homolog |
| 100352955 | XM_002718315 | LOC100352955 | Translocase of outer mitochondrial membrane 20 homolog (yeast)-like |
| 100341031 | XM_002717348 | LOC100341031 | Translocase of outer mitochondrial membrane 20 homolog |
| 100358561 | XM_002721228 | LOC100358561 | Translocase of outer mitochondrial membrane 34 |
| 100349412 | XM_002715147 | LOC100349412 | Translocase of outer mitochondrial membrane 40-like |
| 100352274 | XM_002716612 | TOMM70A | Translocase of outer mitochondrial membrane 70 homolog A (S. cerevisiae) |
| 100009292 | NM_001082404 | TP53 | Tumor protein p53 |
| 100338710 | XM_002723644 | LOC100338710 | Translocator protein-like |
| 100328618 | NM_001171077 | UCP1 | Uncoupling protein 1 (mitochondrial, proton carrier) |
| 100359188 | XM_002708700 | LOC100359188 | Uncoupling protein 2 |
| 100341210 | XM_002719887 | LOC100341210 | Ubiquitously-expressed transcript |

**S1 File Table C: Gene lists for pathway-focused gene expression analysis:**

**PPAR Targets**

| **NCBI Gene ID** | **GenBank** | **Symbol** | **Description** |
| --- | --- | --- | --- |
| 100339733 | XM_002713530 | ACAA2 | Acetyl-CoA acyltransferase 2 |
| 100358521 | XM_002712562 | ACADL | Acyl-CoA dehydrogenase, long chain |
| 100347504 | XM_002715911 | ACADM | Acyl-CoA dehydrogenase, C-4 to C-12 straight chain |
| 100338450 | XM_002722933 | ACOX1 | Acyl-CoA oxidase 1, palmitoyl |
| 103347455 | XM_008253979 | ACOX3 | Acyl-CoA oxidase 3, pristanoyl |
| 100358351 | XM_002712500 | ACSL3 | Acyl-CoA synthetase long-chain family member 3 |
| 100342485 | XM_002720191 | ACSL4 | Acyl-CoA synthetase long-chain family member 4 |
| 100344347 | XM_008270510 | ACSL5 | Acyl-CoA synthetase long-chain family member 5 |
| 100009027 | NM_001082222 | ADIPOQ | Adiponectin, C1Q and collagen domain containing |
| 100009253 | NM_001101687 | APOA1 | Apolipoprotein A-I |
| 100339653 | XM_002715252 | APOA2 | Apolipoprotein A-II |
| 100328796 | NM_001171409 | APOA5 | Apolipoprotein A-V |
| 100342936 | XM_002708371 | APOC3 | Apolipoprotein C-III |
| 100009337 | NM_001082643 | APOE | Apolipoprotein E |
| 100350611 | XM_002707944 | AQP7 | Aquaporin 7 |
| 100008825 | XM_002712016 | CD36 | CD36 molecule (thrombospondin receptor) |
| 100358955 | XM_002715103 | CDKN2C | Cyclin-dependent kinase inhibitor 2C (p18, inhibits CDK4) |
| 100339721 | XM_008257294 | CHD9 | Chromodomain helicase DNA binding protein 9 |
| 100008713 | NM_001082049 | CLU | Clusterin |
| 100353138 | XM_002723234 | CPT1B | Carnitine palmitoyltransferase 1B (muscle) |
| 100346210 | XM_002715617 | CPT2 | Carnitine palmitoyltransferase 2 |
| 100351999 | XM_002712602 | CREB1 | CAMP responsive element binding protein 1 |
| 100340619 | XM_008249593 | CREBBP | CREB binding protein |
| 100348736 | NM_001190430 | CYP27A1 | Sterol 26-hydroxylase, mitochondrial |
| 100328551 | NM_001170929 | CYP7A1 | Cytochrome P450, family 7, subfamily A, polypeptide 1 |
| 100009301 | NM_001082622 | CYP8B1 | Cytochrome P450, family 8, subfamily B, polypeptide 1 |
| 100350630 | XM_008253884 | DGAT1 | Diacylglycerol O-acyltransferase 1 |
| 100344271 | XM_008249087 | ELN | Elastin |
| 100354135 | XM_002721395 | EP300 | E1A binding protein p300 |
| 100339351 | XM_002721596 | ETFA | Electron-transfer-flavoprotein, alpha polypeptide |
| 100340346 | XM_002720545 | ETFDH | Electron-transferring-flavoprotein dehydrogenase |
| 100354672 | XM_002709637 | FABP1 | Fatty acid binding protein 1, liver |
| 100354372 | XM_002717226 | FABP2 | Fatty acid binding protein 2, intestinal |
| 100348000 | XM_002716060 | FABP3 | Fatty acid binding protein 3, muscle and heart (mammary-derived growth inhibitor) |
| 100009416 | XM_002710655 | FABP4 | Fatty acid binding protein 4, adipocyte |
| 100358103 | XM_002714798 | FABP7 | Fatty acid binding protein 7, brain |
| 100352712 | XM_002721017 | FADS2 | Fatty acid desaturase 2 |
| 100342557 | XM_002716117 | FGR | Feline Gardner-Rasheed sarcoma viral oncogene homolog |
| 100342099 | XM_002708648 | LOC100342099 | Frizzled-4-like |
| 100354726 | XM_008272484 | GK | Glycerol kinase |
| 100354726 | XM_008272484 | GK | Glycerol kinase |
| 100009579 | NM_001082782 | HIF1A | Hypoxia inducible factor 1, alpha subunit (basic helix-loop-helix transcription factor) |
| 100349912 | XM_002712368 | HSPD1 | Heat shock 60kDa protein 1 (chaperonin) |
| 100355759 | XM_002708766 | ILK | Integrin-linked kinase |
| 100338474 | XM_002710589 | KLF10 | Kruppel-like factor 10 |
| 100303762 | XM_002713987 | LIFR | Leukemia inhibitory factor receptor alpha |
| 100346262 | XM_002710078 | LPIN1 | Lipin 1 |
| 100340171 | NM_001177330 | LPL | Lipoprotein lipase |
| 100354064 | XM_008271398 | MED1 | Mediator complex subunit 1 |
| 100338228 | XM_002711711 | MLYCD | Malonyl-CoA decarboxylase |
| 100009110 | NM_001171139 | MMP1 | Matrix metallopeptidase 1 (interstitial collagenase) |
| 100008993 | NM_001082203 | MMP9 | Matrix metallopeptidase 9 (gelatinase B, 92kDa gelatinase, 92kDa type IV collagenase) |
| 100356750 | XM_008274804 | NCOA3 | Nuclear receptor coactivator 3 |
| 100356436 | XM_002710828 | NCOA6 | Nuclear receptor coactivator 6 |
| 100352900 | NM_001184956 | NR1H3 | Nuclear receptor subfamily 1, group H, member 3 |
| 100009322 | NM_001082633 | OLR1 | Oxidized low density lipoprotein (lectin-like) receptor 1 |
| 100355682 | XM_002724299 | PCK1 | Phosphoenolpyruvate carboxykinase 1 (soluble) |
| 100144341 | XM_008269396 | PCK2 | Phosphoenolpyruvate carboxykinase 2 (mitochondrial) |
| 100347954 | XM_002723512 | LOC100347954 | 3-phosphoinositide dependent protein kinase-1 |
| 100008882 | NM_001082141 | PLTP | Phospholipid transfer protein |
| 100356422 | XM_002723354 | PPARA | Peroxisome proliferator-activated receptor alpha |
| 100008615 | NM_001082000 | PPARD | Peroxisome proliferator-activated receptor delta |
| 100008892 | NM_001082148 | PPARG | Peroxisome proliferator-activated receptor gamma |
| 100349219 | XM_002709377 | PPARGC1A | Peroxisome proliferator-activated receptor gamma, coactivator 1 alpha |
| 100340477 | XM_002710161 | PPARGC1B | Peroxisome proliferator-activated receptor gamma, coactivator 1 beta |
| 100339175 | XM_008270397 | PPRC1 | Peroxisome proliferator-activated receptor gamma, coactivator-related 1 |
| 100358716 | XM_002718493 | PTEN | Phosphatase and tensin homolog |
| 100348019 | XM_002719177 | PYY | Peptide YY |
| 100354287 | XM_002714483 | RXRB | Retinoid X receptor, beta |
| 100009012 | XM_002715868 | RXRG | Retinoid X receptor, gamma |
| 100347254 | XM_002716284 | LOC100347254 | Arylacetamide deacetylase |
| 100349271 | XM_002718460 | SIRT1 | Sirtuin 1 |
| 100358417 | XM_002710091 | SLC22A5 | Solute carrier family 22 (organic cation/carnitine transporter), member 5 |
| 100352079 | XM_002713398 | SLC25A20 | Solute carrier family 25 (carnitine/acylcarnitine translocase), member 20 |
| 100352217 | XM_002722970 | SLC27A4 | Solute carrier family 27 (fatty acid transporter), member 4 |
| 100340287 | XM_002721897 | SLC27A5 | Solute carrier family 27 (fatty acid transporter), member 5 |
| 100349387 | XM_002710184 | SLC27A6 | Solute carrier family 27 (fatty acid transporter), member 6 |
| 100345769 | XM_002715057 | SMARCD3 | SWI/SNF related, matrix associated, actin dependent regulator of chromatin, subfamily d, member 3 |
| 100353957 | XM_008270242 | SORBS1 | Sorbin and SH3 domain containing 1 |
| 100341599 | XM_008256012 | SRC | V-src avian sarcoma (Schmidt-Ruppin A-2) viral oncogene homolog |
| 100339300 | XM_008255539 | TGS1 | Trimethylguanosine synthase 1 |
| 100346106 | XM_002712835 | TNFRSF1A | Tumor necrosis factor receptor superfamily, member 1A |
| 100355951 | XM_002715643 | TXNIP | Thioredoxin interacting protein |
| 100328618 | NM_001171077 | UCP1 | Uncoupling protein 1 (mitochondrial, proton carrier) |


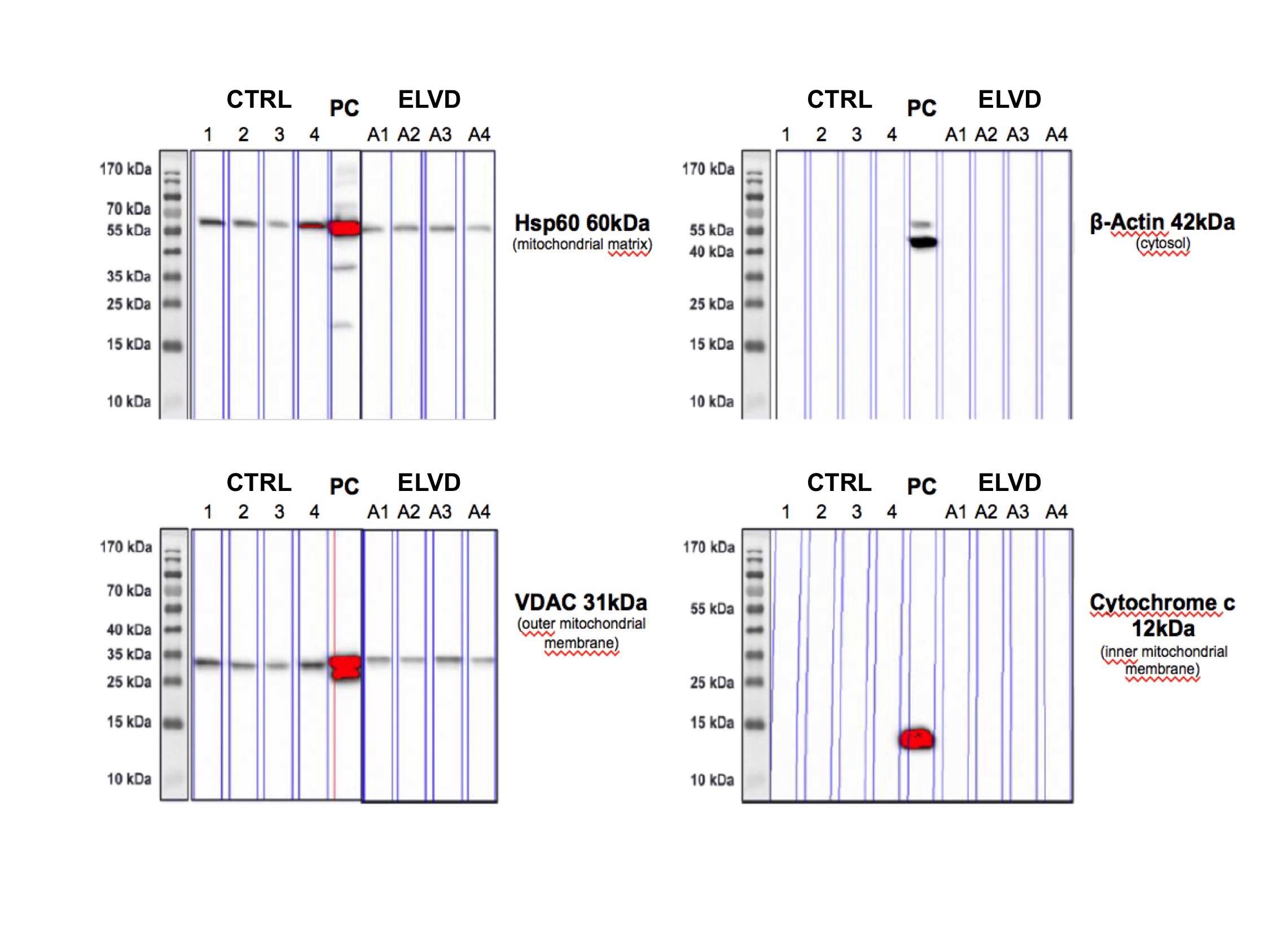
**S1 File Figure A: Mitochondria were sufficiently isolated from whole LM tissue**

**A**


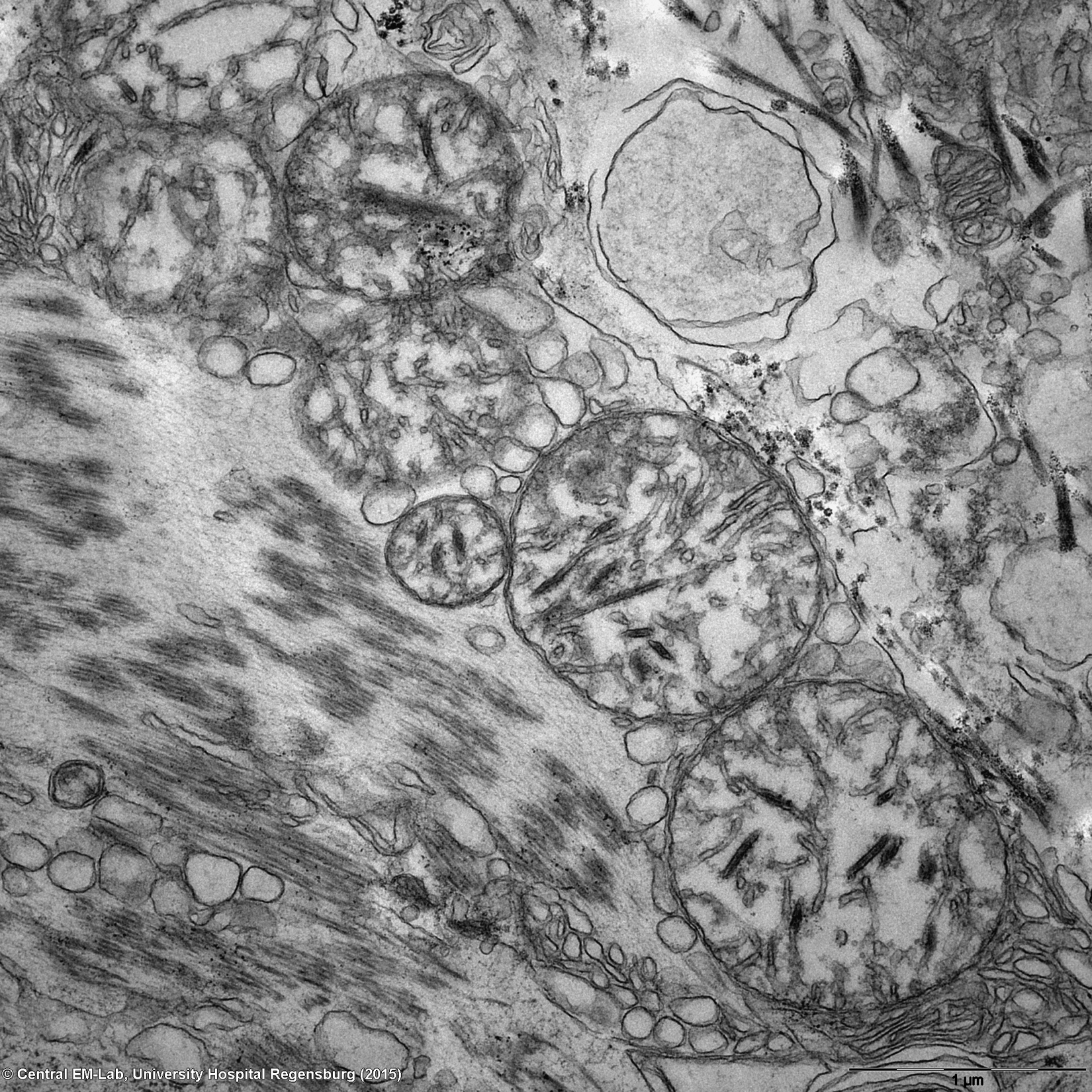


**B**
